# A gene-derived SNP-based high resolution linkage map of carrot including the location of QTL conditioning root and leaf anthocyanin pigmentation

**DOI:** 10.1186/1471-2164-15-1118

**Published:** 2014-12-16

**Authors:** Pablo F Cavagnaro, Massimo Iorizzo, Mehtap Yildiz, Douglas Senalik, Joshua Parsons, Shelby Ellison, Philipp W Simon

**Affiliations:** Department of Horticulture, University of Wisconsin-Madison, 1575 Linden Drive, Madison, WI 53706 USA; CONICET, Facultad de Ciencias Agrarias – Universidad Nacional de Cuyo, and INTA E.E.A. La Consulta, Ex Ruta 40. km 96, La Consulta CC 8, Mendoza, 5567 Argentina; Department of Agricultural Biotechnology, Faculty of Agriculture, Yuzuncu Yil University, 65080 Van, Turkey; USDA-Agricultural Research Service, Vegetable Crops Unit, University of Wisconsin-Madison, 1575 Linden Drive, Madison, WI 53706 USA

**Keywords:** Carrot, Anthocyanins, QTL mapping, Linkage map, Single nucleotide polymorphism

## Abstract

**Background:**

Purple carrots accumulate large quantities of anthocyanins in their roots and leaves. These flavonoid pigments possess antioxidant activity and are implicated in providing health benefits. Informative, saturated linkage maps associated with well characterized populations segregating for anthocyanin pigmentation have not been developed. To investigate the genetic architecture conditioning anthocyanin pigmentation we scored root color visually, quantified root anthocyanin pigments by high performance liquid chromatography in segregating F_2_, F_3_ and F_4_ generations of a mapping population, mapped quantitative trait loci (QTL) onto a dense gene-derived single nucleotide polymorphism (SNP)-based linkage map, and performed comparative trait mapping with two unrelated populations.

**Results:**

Root pigmentation, scored visually as presence or absence of purple coloration, segregated in a pattern consistent with a two gene model in an F_2_, and progeny testing of F_3_-F_4_ families confirmed the proposed genetic model. Purple petiole pigmentation was conditioned by a single dominant gene that co-segregates with one of the genes conditioning root pigmentation. Root total pigment estimate (RTPE) was scored as the percentage of the root with purple color.

All five anthocyanin glycosides previously reported in carrot, as well as RTPE, varied quantitatively in the F_2_ population. For the purpose of QTL analysis, a high resolution gene-derived SNP-based linkage map of carrot was constructed with 894 markers covering 635.1 cM with a 1.3 cM map resolution. A total of 15 significant QTL for all anthocyanin pigments and for RTPE mapped to six chromosomes. Eight QTL with the largest phenotypic effects mapped to two regions of chromosome 3 with co-localized QTL for several anthocyanin glycosides and for RTPE. A single dominant gene conditioning anthocyanin acylation was identified and mapped.

Comparative mapping with two other carrot populations segregating for purple color indicated that carrot anthocyanin pigmentation is controlled by at least three genes, in contrast to monogenic control reported previously.

**Conclusions:**

This study generated the first high resolution gene-derived SNP-based linkage map in the Apiaceae. Two regions of chromosome 3 with co-localized QTL for all anthocyanin pigments and for RTPE, largely condition anthocyanin accumulation in carrot roots and leaves. Loci controlling root and petiole anthocyanin pigmentation differ across diverse carrot genetic backgrounds.

**Electronic supplementary material:**

The online version of this article (doi:10.1186/1471-2164-15-1118) contains supplementary material, which is available to authorized users.

## Background

Anthocyanins, a subclass of water-soluble colored flavonoids, provide red, blue and purple pigmentation to different organs of a wide range of higher plants [1]. These plant pigments play important roles, such as attraction of pollinators and seed dispersers and, given their antioxidant properties, protection against ultraviolet (UV) and high intensity light, drought, wounding, cold temperatures and phytopathogen attack [2–5]. In addition, the consumption of anthocyanin-rich fruits and vegetables is implicated to confer a number of health-related benefits, including protection against oxidative stress, coronary heart disease, inflammation, some types of cancer and other age-related diseases [6]. However, the health effects of dietary anthocyanins depend on amounts consumed and on their bioavailability. Previous studies suggest that the bioavailability and excretion of anthocyanins and other polyphenols is highly influenced by their chemical structure [7], including the nature of the sugar conjugate, the phenolic aglycone and acylation.

Anthocyanins are synthesized via the flavonoid pathway, a late branch of the shikimic acid pathway [8]. Although the biosynthetic pathways in different anthocyanin-containing species share a majority of common reactions, there are important differences between the types of anthocyanins produced and accumulated by each species. For example, bilberry (*Vaccinium myrtillus*) fruits accumulate a balanced mixture of five major anthocyanin aglycones (delphinidin, petunidin, cyanidin, peonidin and malvidin) and lack pelargonidin [9], whereas snapdragon and maize are incapable of producing delphinidin [10] and carrot (*Daucus carota*) almost exclusively accumulates cyanidin derivatives [11, 12] . Regardless of the species, two classes of genes are involved in anthocyanin biosynthesis: structural genes encoding the enzymes that directly participate in the formation of anthocyanin pigments, and regulatory genes that control the transcription of structural genes. Many of the genes involved in anthocyanin biosynthesis, both structural and regulatory, have been identified and characterized for several model species, such as petunia (*Petunia hybrida*), snapdragon (*Antirrhinum majus*) and maize (*Zea mays*), and both regulatory and structural genes vary widely across species [3]. Consequently, information regarding the genetic control of anthocyanin biosynthesis may not be reliably extrapolated across species.

The genetics underlying anthocyanin pigmentation has been most extensively studied in flowers, fruit and leaves. Only a few reports of genetic control of anthocyanin biosynthesis are published for underground organs –tubers, bulbs and storage roots [13–18].

Anthocyanins may undergo a series of chemical modifications including glycosylation, acylation and methylation. These changes are usually performed by glycosyl-, acyl- and methyl-transferase enzymes, respectively. Several studies have indicated that these modifications produce enormous chemical diversity (reviewed by Pojer et al. [19]) which influence anthocyanin stability and bioavailability. For example, clinical human feeding studies using raw and cooked purple carrots revealed that, compared to the anthocyanins in carrots consumed, the percentage recovery of non-acylated anthocyanins in serum was significantly greater than acylated anthocyanins, suggesting that acylation reduces anthocyanin bioavailability [11, 20–22]. Conversely, acylated anthocyanins are more stable than non-acylated anthocyanins, providing the former with an advantage for their use as colorants in the food industry [19, 21, 23]. Despite their importance, the genetic factors controlling these chemical modifications are still relatively unexplored.

**Figure 1:**
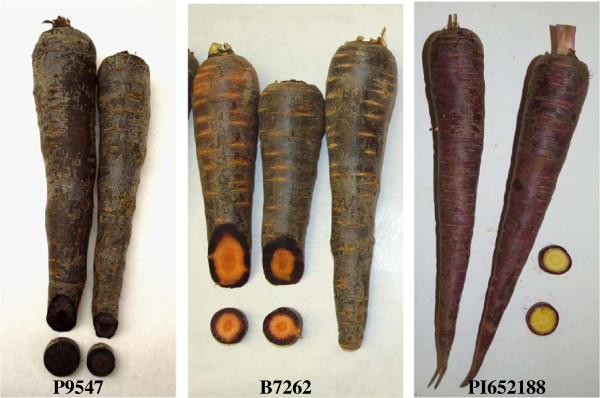
**Purple root phenotypes of three diverse carrot parental genetic stocks.** Root phenotypes of the purple root color source progenitors of carrot mapping populations 70349 (P9547), 10117 (B7262), and 2170 (PI652188), used for comparative mapping of loci controlling anthocyanin pigmentation in root and leaves.

Carrot is a species that can accumulate large quantities of anthocyanins in its storage roots (up to 17–18 mg/100 g fresh weight) [12]. Purple or “black” carrots accumulate almost exclusively cyanidin glycosides, both acylated and non-acylated, with five cyanidin pigments reported in most studies [11, 24] (Table 1). Substantial variation in anthocyanin profiles and in total anthocyanin concentration, and low amounts of derivatives of anthocyanidins other than cyanidin, have been reported among carrot cultivars [12]. In addition, tissue distribution of root purple pigmentation varies greatly across carrot genotypes, ranging from a few pigmented cell layers in the periderm to a completely- and intensively-colored root (Figure 1). The significance of dietary anthocyanins in human health and the extensive natural variation for the above traits has attracted the attention of carrot breeders. Current breeding programs in purple carrot aim at increasing total anthocyanin content as well as achieving favorable ratios of acylated *versus* non-acylated anthocyanins depending on the end-market purpose, with a preference for high content of acylated anthocyanins for their use as food colorants, but conversely, high level of non-acylated forms for increasing bioavailability and nutraceutical value.

**Table 1:** **Carrot cyanidin derivatives with approximate HPLC retention times and molecular masses**

Compound	Abbreviation	RT	MW
Cy-3-(2”-xylose-6-glucose-galactoside)	Cy3XGG	14.0	743
Cy-3-(2”-xylose-galactoside)	Cy3XG	15.1	581
Cy-3-(2”-xylose-6”-sinapoyl-glucose-galactoside)	Cy3XSGG	15.4	949
Cy-3-(2”-xylose-6”-feruloyl-glucose-galactoside	Cy3XFGG	16.0	919
Cy-3-(2”-xylose-6”-(4-coumuroyl)glucose-galactoside)	Cy3XCGG	16.4	889

RT is retention time (min) for the chromatographic procedure described in the Materials and Methods section. MW is molecular weight.

To date, a single dominant gene controlling anthocyanin accumulation in carrot roots, *P*_*1*_, has been described [17]. Recently, *P*_*1*_ was genetically mapped, as were several anthocyanin biosynthetic genes (five structural and three regulatory genes), but no tight linkage was found between *P*_*1*_ and any of the candidate structural genes evaluated [18]. However, the five structural genes (*CHS*_*1*_*, DFR*_*1*_*, F3H, LDOX*_*2*_*, PAL*_*3*_*)* were expressed differentially and in decreasing order among those listed when comparing solid-purple (purple in phloem and xylem), purple orange (purple phloem and orange xylem) and orange carrots, with transcript accumulation coinciding with anthocyanin accumulation. These studies suggested a coordinated regulatory control of anthocyanin biosynthesis in the carrot root, but the molecular and biochemical basis of genetic factors controlling the presence vs. absence of carrot root anthocyanin pigmentation remain unknown. Furthermore, very little is known about the genetics of anthocyanin accumulation in carrot organs and tissues other than the tap root. Variable expression of purple pigmentation in leaves, nodes and flowers was reported but not genetically characterized, and a simply inherited dominant locus controlling purple *versus* green pigmentation in petioles, *P*_*2*_, was also described [17] and linkage between *P*_*1*_ and *P*_*2*_ was suggested but not mapped.

Phenotypic evaluations of *P*_*1*_ and *P*_*2*_ in previous studies of carrot were performed on the basis of visually scoring for presence or absence of purple color in the root and petiole, without providing a quantitative measurement on the extent of purple pigmentation in these organs. In addition to presence or absence of pigmentation, anthocyanin biosynthetic genes can also modify the profile of pigments and tissue-specific accumulation [3, 25] and to date, few studies in carrot have explored these factors affecting the relative accumulation of different anthocyanin pigments in purple-colored tissues. One exception is a study of variation in pigment profiles of four commercial carrot cultivars which found more than 10-fold variation in total anthocyanin content and significant variation in percentage cyanidin-3-(2’-xylose-galactoside) and cyanidin-3-(2’-xylose-6’-sinapoyl-glucose-galactoside) relative to the most abundant compound they found, cyanidin-3-(2’-xylose-6’-feruloyl-glucose-galactoside [12]. Understanding the genetic architecture conditioning both pigment concentration and the relative accumulation of different anthocyanin glycosides in the carrot root would be of great value for carrot breeding and genetics. Furthermore, since mapping of *P*_*1*_ was performed in a single genetic background, comparative analysis of the loci controlling anthocyanin pigmentation across diverse carrot backgrounds would be of interest, considering the broad variation for phenotype, genotype and geographical origin observed among purple carrot genetic stocks [17].

High density linkage maps constructed with informative sequence-based markers, such as SNPs and SSRs, are essential for fine mapping of QTL, comparative analysis of synteny, searching for candidate genes, facilitating genome sequence assembly, and for marker assisted breeding. The majority of the carrot maps constructed to date were unsaturated and used anonymous dominant markers, such as amplified fragment length polymorphisms and randomly amplified polymorphic DNAs, although in some cases a few codominant SSR or restriction fragment length polymorphisms markers were included [18, 26–32]. A DArT map with the highest map resolution (1.1 cM) achieved to date in carrot was recently reported by Grzebelus et al. [33]. However, the anonymous and dominant nature of DArT markers do not allow straight forward comparative map analysis or identification of candidate genes associated with QTL.

In the current study, we developed a SNP-based linkage map with an unprecedented high map resolution in entire Apiaceae family, using a carrot population segregating for anthocyanin pigmentation in root and leaves. Through detailed phenotyping including HPLC analysis we detected and mapped significant QTL for the different root anthocyanin glycosides. Their map position relative to the position of a large-effect QTL for root pigment content revealed two regions on chromosome 3 largely conditioning purple root pigmentation and anthocyanin acylation, respectively. In addition, linkage mapping of the locus controlling purple pigmentation in leaves was performed, with its position being tightly linked to one of the QTL for root anthocyanins. A similar genetic control for purple pigmentation was found in an unrelated (Chinese) genetic background. Finally, we mapped the genetic loci controlling both root and petiole purple pigmentation in three unrelated carrot backgrounds and discovered that root and petiole anthocyanin pigmentation in diverse genetic backgrounds mapped to not only *P*_*1*_ but also other loci.

## Results

### Inheritance of purple root and petiole color

In population 70349, a total of 519 F_2_ plants were grown in two contrasting environments and phenotyped for presence/absence of purple pigmentation in carrot roots. In the largest 70349 subset (*N* = 497), evaluated under greenhouse conditions, purple pigmentation deviated significantly (*P* < 0.001) from the 3:1 ratio expected for a single dominant gene in an F_2_, that was reported previously for *P*_*1*_ in other carrot backgrounds [17, 18, 26] (Table 2). A smaller subset (*N* = 22) of field-grown 70349 plants also deviated from the expected 3:1 ratio (χ^2^ = 4.91, *P* < 0.03). Additional analyses of the data considering other genetic models revealed a good fit for a 9:7 segregation ratio in both greenhouse (χ2 = 0.003, P = 0.96) and field-grown (χ2 = 0.03, P = 0.87) plants of 70349. These data suggest that two dominant loci interact epistatically in the genetic control of root purple pigmentation in the 70349 background.

**Table 2:** **Segregation of purple root pigmentation in carrot F**_**2**_ **families 70349, 2170, and 10117, and in F**_**3**_ **and F**_**4**_ **families derived from population 70349**

Purple root source	Generation ^†^	Phenotype of parent selfed plant ^ζ^	Number of progeny					
			Purple	Non-purple	Total	Expected seg. ratio ^β^	χ ^2^	***P***
P9547	F_2_ (70349) - gh	P	279	218	497	3:1	94.3*	<0.001
						9:7	0.003	0.96
	F_2_ (70349) -f	P	12	10	22	3:1	4.91*	0.03
						9:7	0.03	0.87
	F_3_ (9534) - f	P	77	58	135	1:0, **9:7**, 3:1	0.03	0.85
	F_3_ (9535) - f	P	62	53	115	1:0, **9:7**, 3:1	0.26	0.61
	F_3_ (9553) - f	P	20	17	37	1:0, **9:7**, 3:1	0.07	0.79
	F_3_ (9555) - f	P	28	29	57	1:0, **9:7**, 3:1	1.18	0.28
	F_3_ (9556) - f	P	18	13	31	1:0, **9:7**, 3:1	0.04	0.84
	F_3_ (9557) - f	P	73	61	134	1:0, **9:7**, 3:1	0.17	0.68
	F_3_ (9561) - f	P	70	54	124	1:0, **9:7**, 3:1	0.00	0.96
	F_3_ (9562) - f	P	40	30	70	1:0, **9:7**, 3:1	0.02	0.88
	F_3_ (9567) - f	P	32	30	62	1:0, **9:7**, 3:1	0.54	0.46
	F_3_ (9571) - f	P	38	41	79	1:0, **9:7**, 3:1	2.13	0.14
	F_3_ (8598) - gh	P	11	9	20	1:0, **9:7**, 3:1	0.01	0.91
	F_3_ (8599) - gh	P	20	15	35	1:0, **9:7**, 3:1	0.01	0.92
	F_3_ (9563) - gh	P	35	42	77	1:0, **9:7**, 3:1	3.65	0.06
	F_3_ (8596) - gh	P	19	0	19	**1:0**, 9:7, 3:1	0.00	0.59
	F_3_ (9536) - f	P	30	12	42	1:0, 9:7, **3:1**	0.29	0.72
	F_3_ (9539) - f	P	8	2	10	1:0, 9:7, **3:1**	0.13	0.91
	F_3_ (9551) - f	P	83	27	110	1:0, 9:7, **3:1**	0.01	0.73
	F_3_ (9564) - f	P	18	7	25	1:0, 9:7, **3:1**	0.12	0.64
	F_4_ (2313) - gh	P	19	5	24	1:0, 9:7, **3:1**	0.22	0.59
	F_3_ (9533) - f	NP	0	75	75	0:1	0.00	1.00
	F_3_ (9538) - f	NP	0	51	51	0:1	0.00	1.00
	F_3_ (9540) - f	NP	0	24	24	0:1	0.00	1.00
	F_3_ (9541) - f	NP	0	62	62	0:1	0.00	1.00
	F_3_ (9543) - f	NP	0	39	39	0:1	0.00	1.00
	F_3_ (9544) - f	NP	0	74	74	0:1	0.00	1.00
	F_3_ (9546) - f	NP	0	65	65	0:1	0.00	1.00
	F_3_ (9547) - f	NP	0	109	109	0:1	0.00	1.00
	F_3_ (9548) - f	NP	0	46	46	0:1	0.00	1.00
	F_3_ (9549) - f	NP	0	88	88	0:1	0.00	1.00
	F_3_ (9550) - f	NP	0	52	52	0:1	0.00	1.00
	F_3_ (9552) - f	NP	0	20	20	0:1	0.00	1.00
	F_3_ (9554) - f	NP	0	60	60	0:1	0.00	1.00
	F_3_ (9560) - f	NP	0	78	78	0:1	0.00	1.00
	F_3_ (9565) - f	NP	0	80	80	0:1	0.00	1.00
	F_3_ (9566) - f	NP	0	57	57	0:1	0.00	1.00
	F_3_ (9568) - f	NP	0	49	49	0:1	0.00	1.00
	F_3_ (9569) - f	NP	0	77	77	0:1	0.00	1.00
	F_3_ (9570) - f	NP	0	42	42	0:1	0.00	1.00
	F_3_ (9572) - f	NP	0	48	48	0:1	0.00	1.00
	F_3_ (9573) - f	NP	0	59	59	0:1	0.00	1.00
	F_3_ (9574) - f	NP	0	76	76	0:1	0.00	1.00
	F_3_ (8593) - gh	NP	0	32	32	0:1	0.00	1.00
	F_3_ (8597) - gh	NP	0	15	15	0:1	0.00	1.00
	F_3_ (8600) - gh	NP	0	30	30	0:1	0.00	1.00
	F_4_ (2304) - gh	NP	0	23	23	0:1	0.00	1.00
	F_4_ (2307) - gh	NP	0	42	42	0:1	0.00	1.00
PI652188	F_2_ (2170)	P	49	16	65	3:1	0.01	0.94
B7262	F_2_ (10117)	P	59	16	75	3:1	0.54	0.46

^†^Generation followed by the population name, in parenthesis, and the cultivation environment where ‘f’ denotes field-grown plants at El Centro, California; ‘gh’ denotes greenhouse conditions (University of Wisconsin). ^ζ^Root phenotype was either purple (P) or non-purple (NP). ^β^When multiple segregation ratios were considered, based on the progenitor phenotype, the χ^2^ tested is indicated in bold. *P < 0.05.

Further segregation analysis of purple root color in F_3_ and F_4_ derivatives of 70349 revealed a general agreement with the genetic model proposed based upon segregation ratios observed in the F_2_. Under the proposed model, derivative families of purple rooted carrots are expected to segregate in purple: non-purple ratios of 1:0 (if the progenitor is *AABB*), 9:7 (if the progenitor is *AaBb*) or 3:1 (if the progenitor is either *AABb* or *AaBB*), whereas all derivatives from non-purple rooted carrots (with genotype *A_bb*, *aaB_*, *aabb*) are expected to be 100% non-purple, since both loci at a dominant state are required for pigment accumulation. Of the 46 70349-derived populations analyzed, 19 were developed from self-pollinating single purple-rooted plants and 27 populations derived from non-purple rooted progenitors (Table 2).

All population 70349 derived F_3_s and F_4_s fit expected ratios for the two-gene model proposed for the genetic control of root purple pigmentation (Table 2). Among the 19 populations derived from purple carrots, 13 F_3_s segregated for ‘purple: non-purple’ in a 9:7 ratio, 5 populations (four F_3_s and one F_4_) showed a 3:1 ratio, and one F_3_ was fixed for purple (1:0). A good fit was observed among F_3_ families for the various expected ratios (ranging from χ^2^ = 0.00 to 3.65; *P* = 0.06 to 1.00) (mean χ^2^ = 0.46, *P* = 0.66). As expected, all the progenies derived from non-purple carrots (25 F_3_s and 2 F_4_s) were 100% non-purple rooted (χ^2^ = 0.00, *P* = 1.00).

In population 70349, anthocyanin pigmentation in petioles segregated as a simply inherited trait, with purple being dominant over green (χ^2^ = 2.27, *P* = 0.14). Among the 90 plants with non-purple petioles, all but two had non-purple roots. Were it not for those two exceptions, these data would indicate that one of the two genes responsible for purple root pigmentation also conditioned purple petioles. If those two exceptions are due to variable penetrance, that model would be valid, but the two exceptions could also indicate that the gene conditioning petiole pigmentation is very closely linked to one of the genes for root pigmentation.

Anthocyanin pigmentation was also evaluated in F_2_ population 2170, genetically unrelated to population 70349 (Table 2). In this population, purple pigmentation was dominant over non-purple and segregated as a single gene in both roots (χ^2^ = 0.01, *P* = 0.94) and petioles (χ^2^ = 0.13, *P* = 0.72). A previous study by Yildiz et al. [18] also found simple inheritance for root purple color in population 10117, an F_2_ with no petiole pigmentation that is unrelated to both the 70349 and 2170 populations. Figure 1 presents root characteristics of the purple source progenitor in these three populations.

### Root pigment analysis

In contrast to the deep pigmentation of P9547 (Figure 1), the purple pigmentation in the 70349 F_1_ and F_2_ populations, derived from P9547, was not as intense and primarily on the root exterior. Root total pigment estimate (RTPE) varied quantitatively in the F_2_ and ranged from 0% (no purple pigmentation) to 100% (root surface completely purple) (Additional file 1) with most purple roots having RTPE values between 10% and 89% (Additional file 2). All plants with dark purple petioles had purple roots while 21.7% of the plants with purple petioles had non-purple roots, and 38.1% of the plants with pale purple petioles had non-purple roots. RTPE values were relatively evenly distributed from 5% to 100% regardless of petiole color intensity (Table 3).

**Table 3:** **Frequency distribution of petiole color, root total pigment estimate (RTPE) and high total acylated anthocyanins in population 70349**

Petiole color	Root total pigment estimate (RTPE)												
	0	5	10	20	30	40	50	60	70	80	90	100	Total
Dark Purple	0	3	9	4	3	4	1	2	2	3	2	1	34
High Σacyl*	-	-	5/5	4/4	2/3	3/3	1/1	2/2	2/2	3/3	2/2	1/1	25/26
Purple	69	21	18	23	24	17	19	21	21	17	10	4	264
High Σacyl	-	5/7	8/13	13/20	14/19	16/17	16/19	14/15	19/21	13/15	8/9	1	128/157
Pale Purple	8	4	4	1	-	-	-	3	1	-	-	-	21
High Σacyl	-	1/4	2/3	1/1	-	-	-	2/3	1/1	-	-	-	7/12

*Ratios reflect number of plants with high % acylated anthocyanins (>50% acylated)/total number of plants evaluated by HPLC for a given RTPE category.

HPLC data for root anthocyanins were obtained from 208 plants of population 70349 with >5% RTPE purple pigmentation. Pigment analyses in F_2_ individuals with 5% or less of their tap root surface colored with purple was not performed due to an insufficient amount of pigmented tissue available for HPLC analysis.

HPLC data demonstrated the accumulation of five cyanidin derivatives in roots of population 70349; two non-acylated (Cy3XG, Cy3XGG) and three acylated pigments (Cy3XFGG, Cy3XSGG, Cy3XCGG) (see Table 1 for complete name of compounds; and chemical structure in Additional file 3). Analysis of frequency distributions for the quantitative data revealed clear bimodal distributions for percentage Cy3XGG and percentage Cy3XFGG, with purple-rooted individuals having either less than 22%, or higher than 60% of Cy3XGG, while RTPE and the other cyanidin derivatives more closely approximated normal distributions (Additional file 2). All individuals with low % Cy3XGG had high % acylated (Cy3XSGG and Cy3XFGG) anthocyanins (in total, 52% to 98%) while all high % Cy3XGG individuals had low % acylated anthocyanins (4% to 24%). In fact, the relative content of the non-acylated Cy3XGG was strongly and inversely correlated with the total relative content of acylated anthocyanins (r = −0.99, P < 0.001), as is evident in the clustering of the relative distributions of these compounds among F_2_ individuals. The bimodal distribution of % Cy3XGG/% acylated was further investigated. Two clear groups of 38 individuals with ‘high Cy3XGG/low acylated’ and 170 individuals with ‘low Cy3XGG/high acylated’ were identified (Additional file 4: Figure S1.A). These results suggest that acylation of Cy3XGG to produce Cy3XSGG and Cy3XFGG results in the shift from ‘low’ to ‘high’ content of acylated anthocyanins and a relatively simple genetic basis underlies this acylation. Total acylated anthocyanin content was not associated with RTPE values (r = 0.20), but plants with dark purple petioles tended to be in the high acylated category (Table 3).

Among carrots included in HPLC analysis, the 38:170 segregation ratio did not fit a single gene model for an F2 (χ2 = 4.48, P = 0.034), with ‘low Cy3XGG/high acylated’ being dominant over ‘high Cy3XGG/low acylated’. This distorted segregation ratio may suggest a more complex genetic basis underlying this trait, or may reflect the fact that HPLC analysis was only performed on a subset of purple roots in the population.

Among individuals with high content of acylated anthocyanins (i.e., those with low Cy3XGG) the feruloyl-containing pigment (Cy3XFGG) was the most abundant anthocyanin in most plants, with a concentration range of 25-85%, followed by Cy3XSGG (range: 9-60%) and Cy3XCGG (range: 0-5%). Since in most plants, Cy3XCGG was detected in trace amounts and therefore its genetic analysis may not be accurate, this pigment was excluded from QTL analysis. More detailed analyses of relationships among the individual anthocyanin pigments and RTPE are in Additional file 4: Figure S1.B and Table S2.

### Construction of the framework linkage map in population 70349

Out of the 4000 SNPs evaluated, 3611 (91%) produced distinct genotypic clusters using the Kbioscience (Hoddesdon, England) platform that functions with the KASPar assay. Of these, 1099 were polymorphic and were used for linkage mapping analysis. Screening of 60 SSR markers with known chromosome location in a subset of the 70349 population allowed the identification of 40 polymorphic SSRs.

**Figure 2:**
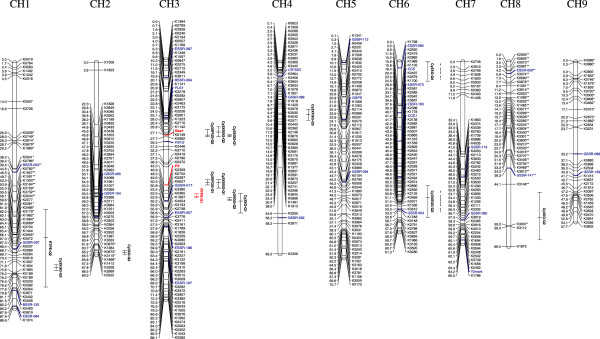
**Carrot genetic linkage map of the 70349 mapping population.** Linkage groups were oriented and named according to their corresponding physical chromosomes (markers in blue indicate anchoring markers with known chromosome location) [34]. Markers in red corresponds to the phenotypic loci controlling purple pigmentation in root (*P*_*3*_) and acylation of root anthocyanins (*Raa1*). Bars to the right of the linkage groups represent support intervals of QTL for individual anthocyanin pigments (Cy3XG, Cy3XGG, Cy3XSGG, Cy3XFGG) and for total root pigment estimate (RTPE). The RTPE QTL with largest phenotypic effects, explaining 50.5% of the variation, is denoted in red on CH3. Asterisks indicate markers that were distorted from expected segregation ratios for P ≤ 0.01 (*) and P ≤ 0.005 (**).

At LOD > 10, of the 1139 polymorphic loci, 894 markers were grouped in nine linkage groups (LGs), consistent with the carrot haploid chromosome number (*n* = 9). All markers with known chromosome location grouped as expected and all groups were unambiguously anchored to the nine carrot chromosomes (Figure 2).

Evaluation of marker order in each linkage group by CheckMatrix revealed minimal or no error in scores, therefore indicating a high quality linkage map (Additional file 5). In order to reduce the complexity of the linkage map figure, redundant markers (i.e., markers sharing exactly the same map position and having no recombination among them) were removed from the image, leaving a single marker per locus or bin. The resulting carrot genetic map includes 482 non-redundant data points (Figure 2). A complete list of all the mapped markers, including redundant markers, is provided in Additional file 6.

Marker distribution across the nine chromosomes (CHs) is presented in Table 4. The number of markers per chromosome ranged from 50 (35 non-redundant) in CH9 to 141 (82 non-redundant) in CH3. The entire map covered 635.1 cM, with map length ranging from 59.7 cM (CH9) to 88.9 cM (CH1). The overall map had an average linkage group size of 70.8 cM and a map resolution of 1.3 cM.

**Table 4:** **Summary of the carrot F**_**2**_ **population 70349 genetic map**

Chr	No. markers	Non redundant markers	Size of LG (cM)	Mean map resolution ^1^ (cM)
1	89	56	88.8	1.6
2	103	47	69.3	1.5
3	141	82	86.1	1.1
4	107	52	69.2	1.3
5	114	65	70.7	1.1
6	128	57	61.3	1.1
7	92	52	65.5	1.3
8	65	36	66.5	1.8
9	50	35	57.7	1.6
Total	894	482	635.1	1.3

^1^Mean map resolution is the average distance between non-redundant markers for a given chromosome.

Three clusters of distorted markers were identified on CH1, CH8 and CH9. A major portion of CH8 had high segregation distortion (p < 0.01). These results are consistent with recent mapping of DArT markers in this same F_2_ population where high segregation distortion of markers in chromosome 8 was found, resulting in the mapping of a very low number of non-distorted markers [33].

The relative content of the predominant non-acylated anthocyanin pigment Cy3XGG, had a clear bimodal distribution in the F_2_ compatible with a simply-inherited dominant trait, and for mapping purposes was scored as a dominant phenotypic marker. As such, for purposes of map construction, individuals with ≥ 60% Cy3XGG (‘high Cy3XGG/low acylated’) were considered as the recessive status (coded as “A”), and those with ≤ 22% (‘low Cy3XGG/high acylated’) as dominant (coded as “C”), based on their relative frequency distribution which nearly fit a 3:1 ratio (170:38) for individuals with ‘≤22% Cy3XGG’: ‘≥60% Cy3XGG’ or ‘low Cy3XGG/high acylated’: ‘high Cy3XGG/low acylated’. Mapping only included 70349 individuals with >5% pigmented roots (RTPE). With this approach, the genetic factor conditioning accumulation of Cy3XGG was mapped to CH3 and co-localized with marker K0149 (Figure 2). Cy3XGG, mapped as a single dominant gene controlling high *versus* low content of acylated anthocyanins, was denoted *Raa1* (for ‘root anthocyanin acylation’) to distinguish it from Cy3XGG content evaluated as a quantitative trait.

### QTL analysis and mapping in population 70349

QTL analysis was carried out for four anthocyanin glycosides (Cy3XG, Cy3XGG, Cy3XSGG and Cy3XFGG) and for total pigment content (RTPE) in roots of 70349. Significant QTL (p < 0.1) were detected for all traits (Table 5). In total, 15 QTL were mapped onto the carrot linkage map (Figure 2). Consistent with the two-gene model observed for root purple color segregation in F_2_-F_4_ families, two QTL were detected for RTPE on chromosomes 3 and 1. The QTL in chromosome 3 had a strong statistical support (LOD = 26.7) and the largest effect on phenotype, explaining 50.5% of the observed variation. The support interval for this large-effect QTL was delimited within a 1.4 cM map region. The other RTPE QTL, located in chromosome 1, had less statistical support (LOD = 3.7) and only accounted for 4.9% of the variation, and its support interval covered a large region (28 cM) of chromosome 1.

**Table 5:** **Summary of QTL for root total pigment estimate (RTPE) and anthocyanin pigments (Cy3XG, Cy3XGG, Cy3XSGG and Cy3XFGG) in the 70349 F**_**2**_ **population**

Trait	QTL ID	Chromosome	Position (cM)	LOD value	1.5 LOD support interval	Nearest marker	% variation explained
**RTPE**	Q1	3	49.1	26.7	48.1-49.5	K2287	50.5
	Q2	1	63.0	3.7	53.0-81.0	K1664	4.9
**Cy3XG**	Q1	3	50.7	14.8	47.9-55.1	GSSR-017	26.4
	Q2	8	56.1	7.2	47.1-64.1	K0973	11.3
	Q3	2	68.5	6.2	67.8-69.3	K2669	9.6
	Q4	6	0	3.6	0-7.0	K1768	5.3
**Cy3XGG**	Q1	3	27.1	104.7	27.0-27.7	*Raa1*/K0149	73.3
	Q2	3	50.1	84.2	49.5-50.4	K0627	36.6
	Q3	1	74.0	71.4	73.0-75.0	K1964	23.4
**Cy3XSGG**	Q1	3	44.1	41.6	43.1-46.1	*P3*	51.7
	Q2	3	25.9	17.5	24.1-27.7	K1323	13.9
	Q3	6	49.0	8.4	30.1-41.5	K2617	5.7
**Cy3XFGG**	Q1	3	44.1	66.8	43.1-45.0	*P3*	59.2
	Q2	3	27.1	38.3	25.1-27.7	K0149	18.9
	Q3	4	19.1	13.9	17.1-21.1	K3591	4.4

The locus controlling anthocyanin pigmentation in leaves mapped to CH3 and was tightly linked (at 1.9 cM) to marker K2309 This trait was earlier noted to be either tightly linked (<5 cM) to one of the genes for RTPE, or it may correspond to the same gene, with the exceptional plants with purple roots and non-purple petioles due to reduced penetrance of petiole pigmentation. An examination of the markers flanking the purple petiole locus for these two exceptional plants indicated alleles for only the purple-rooted parent. This suggests reduced penetrance of petiole pigmentation rather than recombination of a second gene within this region accounting for these exceptions. Based upon this evidence, we surmise that the same gene controls both petiole and root pigmentation, and we name that gene *P*_*3*_. *P*_*3*_ mapped within the 43.1-55.1 cM region harboring four QTL for root anthocyanins, suggesting that this 12 cM region harbors the genetic determinants conditioning both root and petiole pigmentation.

QTL for individual anthocyanin pigments were distributed across six chromosomes (CH1, CH2, CH3, CH4, CH6 and CH8) (Table 4). The number of QTL detected for each pigment ranged from three to four. The phenotypic variation explained by QTL with the largest effect for each trait was –from smallest to largest- 26.4% for Cy3XG, 51.7% for Cy3XSGG, 59.2% for Cy3XFGG and 73.3% for Cy3XGG.

Eight QTL with the highest LOD values (ranging from 14.8 to 104.7) and the largest phenotypic effects were mapped in two regions of chromosome 3. These two regions covered by the QTL support intervals are 24.1-27.7 cM and 43.1-55.1 cM, respectively. The 43.1-55.1 cM region harbors co-localized QTL for all five traits (i.e., the four anthocyanin glycosides and RTPE). Allelic interactions for markers in this region of chromosome 3 were examined (Additional file 7). For all the QTL in this region, the ‘*A*’ allele at the nearest QTL marker had a recessive effect on phenotype. Root total pigment content estimate was highest in heterozygote (*AB*) individuals for marker K0627, the closest marker linked to the RTPE-QTL. Root pigmentation in these heterozygous individuals was on average nearly ten-fold higher (42.6 ± 27.6%; mean ± SD) than in individuals homozygous for the ‘*B*’ allele (4.75 ± 8.75%). These results suggest an over-dominant effect in individuals heterozygous (*AB*) for the “RTPE QTL”.

The 24.1-27.7 cM region of chromosome 3 harbors overlapping QTL for Cy3XSGG, Cy3XFGG and Cy3XGG (Fig. 2 and Table 5). The Cy3XGG QTL had the highest statistical support (LOD = 104.7) and the largest phenotypic effect (73.3%) of all 15 mapped QTL. In addition, the support interval of Cy3XGG overlapped the shortest map distance (0.7 cM) of all QTL. Interestingly, the narrow QTL region that influenced Cy3XGG content co-localized with *Raa1* in the linkage map, supporting the idea that a single chromosomal region may control anthocyanin acylation in carrot roots. For this gene, the dominant allele (*A_*) conditions acylation of anthocyanins [and this results in a low content (<22%) of Cy3XGG (‘low Cy3XGG/high acylated’), the chemical substrate which is acylated] while the recessive (*BB*) genotype conditions low acylation [which results in a relatively high (>60%) content of Cy3XGG] (Additional file 7).

While it was noted that the *Raa1* gene, with a segregation ratio of 38:170, did not fit a single gene model above, an examination of the allelic status of those plants with purple roots that were not included in HPLC analysis (44 plants) observed that 14 of these individuals had homozygous markers for the recessive allele, and 30 had at least one dominant allele. The inclusion of these data in our earlier segregation analysis for *Raa1* did fit a single gene model for an F_2_ (χ^2^ = 2.56; *P* = 0.11), suggesting that anthocyanin acylation is, in fact, a single gene conditioned by *Raa1.*

As expected based upon their co-localization in the linkage map, analysis of allele interactions for Cy3XSGG and Cy3XFGG QTL that mapped in the 24.1-27.7 cM region (Figure 2) coincided with the observed allelic interactions for Cy3XGG. The ‘*A*’ allele at the nearest marker for Cy3XSGG and Cy3XFGG QTL was dominant over the ‘*B*’ allele, where the dominant genotypes (*A_*) had high content of acylated anthocyanins (mainly Cy3XSGG and Cy3XFGG) and the recessive genotypes (*BB*) had low content of acylated pigments (and high level of the non-acylated Cy3XGG). To account for quantitative variation in these two pigments, the phenotypic cutoff for the dominant (*A_*) and recessive (*BB*) genotypic classes was ~10% for Cy3XSGG (i.e., all ‘*A_*’ individuals had >10% and all *BB* individuals had <10% of this pigment), and ~26% for Cy3XFGG (‘*A_*’: >26% Cy3XFGG; ‘*BB*’: <26% Cy3XFGG) (Additional file 7).

### Comparative mapping of anthocyanin traits

In addition to the 70349 mapping population, the phenotypic traits controlling purple root and petiole pigmentation, and several common markers (SNPs, SSRs) and genes (*FLS*, *F3H*, *PSY2*) were mapped in two other populations, 10117 and 2170. Both developed from crosses between purple- and orange-rooted carrots, where the purple progenitor was genetically and phenotypically different from the genetic source of purple pigmentation in population 70349 and from each other (see Methods Figure 1).

**Figure 3:**
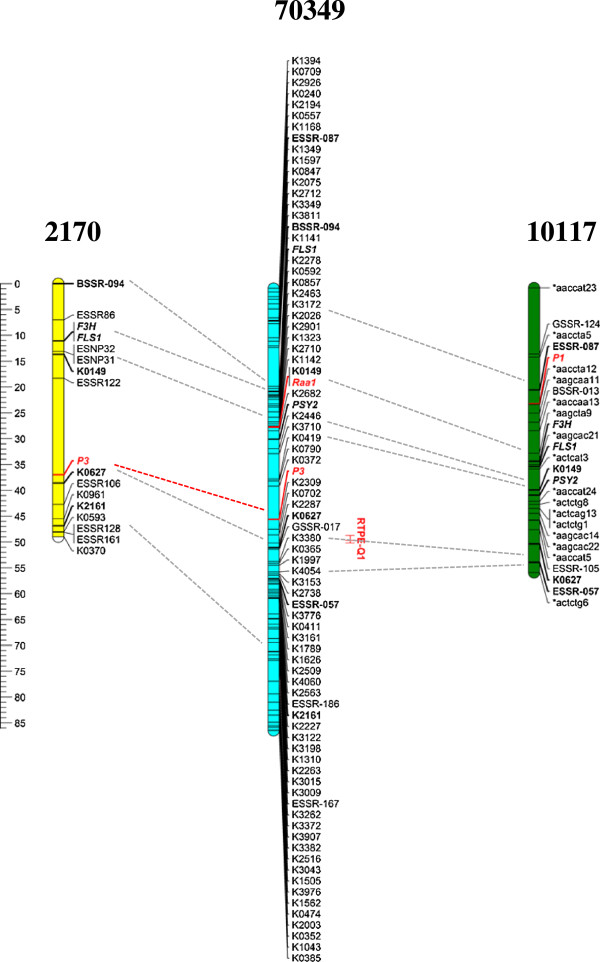
**Comparative mapping of loci on chromosome 3 controlling anthocyanin pigmentation in three carrot genetic backgrounds.** The F_2_ mapping populations 70349, 2170 and 10117 were developed using different purple-rooted genotypes as progenitors. All linkage groups correspond to CH3 of the respective maps. Anthocyanin traits, indicated in red and italic font, include the loci controlling purple root and petiole pigmentation in populations 70349 and 2170 (*P*_*3*_), root pigmentation in 10117 (*P*_*1*_), and *Raa1*, the locus conditioning root anthocyanin acylation in population 70349. The support interval for the QTL for root total pigment estimate (RTPE) in population 70349 is indicated in red. Common markers across CHs are denoted in bold and connected by horizontal dotted lines. The following genes are denoted in italics: *F3H* (flavanone 3-hydroxylase), *FLS* (flavanone 3'hydroxylase), *PSY2* (phytoene synthase-2).

Comparative map analysis of CH3 from 70349, 2170 and 10117 was performed (Figure 3). The use of six data-points in common across the three maps, including markers tightly linked (<5 cM) to all the anthocyanin traits mapped in each chromosome allowed accurate map comparisons and revealed high marker collinearity across the three maps.

In population 10117, purple root pigmentation was previously found to be conditioned by a single dominant gene, *P*_*1*_, [17] which mapped to CH3 [18]. The SNP marker K0627 tightly linked to *P*_*3*_ was included in the 43.1-55.1 cM region of chromosome 3 in population 70349, and was also tightly linked to four co-localized QTL for anthocyanin glycosides. This region (i.e., marker K0627) also mapped to CH3 of population 10117 but was relatively distant (27.3 cM) from *P*_*1*_ (Figure 3). In addition, two markers (K0149 and *PSY2*) in chromosome 3 of population 70349 that were tightly linked to *Raa1*, the locus controlling anthocyanin acylation, and to three overlapping QTL for acylated and non-acylated anthocyanin pigments mapped to CH3 of population 10117 at a distance of 16.8-17.8 cM away from *P*_*1*_. These data clearly indicated that the loci controlling anthocyanin pigmentation in roots of diverse genetic backgrounds differ, and those in the 70349 population are different from *P*_*1*._

In population 2170 the F_1_ plants were all purple-rooted and had purple petioles, whereas in the F_2_, purple pigmentation segregation was consistent with a single gene model (χ^2^ = 0.01, *P* = 0.94) with dominance for purple over non-purple.. Root and petiole pigmentation completely co-segregated in this population, indicating that the same gene controls pigmentation in both organs. This gene mapped with 16 molecular markers (6 SSRs, 8 SNPs and 2 gene-specific markers), conforming a linkage group of 49 cM, with an average distance between adjacent markers of 2.45 cM (Figure 3). The use of chromosome-anchoring markers [34] allowed the orientation and assignment of this LG to carrot chromosome 3.

In CH3 of 2170, the locus controlling purple pigmentation in roots and petioles was tightly linked to K0627, the same marker that was tightly linked to *P*_*3*_, RTPE and four other anthocyanin QTL in 70349, suggesting that the same chromosome region controls root and petiole anthocyanin pigmentation in 2170 and 70349, and this region clearly differs from *P*_*1*_. Thus, we surmise that the locus controlling root purple and petiole pigmentation in 2170 was *P*_*3*_ (Figure 3).

## Discussion

### Development of the first high resolution gene-derived SNP-based linkage map in the Apiaceae

The development of transcriptome-base SNP markers from *D. carota* next-generation sequencing data [35] has opened the possibility for performing cost-effective high-throughput genotyping of carrot mapping and breeding populations. From an initial screening of 4000 SNP markers developed from Expressed Sequence Tag (EST) data [35], we constructed a carrot linkage map with map resolution for a sequence-based marker map unprecedented in the Apiaceae. The resulting genetic map harbored 895 polymorphic molecular markers covering 635.1 cM, with an average map resolution of 1.3 cM. To date, only 117 markers with known sequence information have been mapped in carrot [18, 30, 31] with the most dense map containing 73 sequence-based markers (mainly SSRs) and a resolution of 7.4 cM [30]. Other maps were mainly based on anonymous dominant AFLP, RAPD, RFLP and DArT markers [26, 28, 33]. The total length of the map (635.1 cM) was similar to the map length found in other studies (534.4 cM [26]; 668.7 cM [31]) but smaller than the linkage maps developed by Cavagnaro et al. [30] (1180 – 1273.2 cM), Just et al. [29] (1055–1210.5 cM), Grzebelus et al. [32] (1297.9 - 1504.2 cM). The observed differences in total map length among these studies are most likely attributable to the different software and algorithms used for linkage analysis. While the present study, as well as those of Vivek and Simon [26] and Alessandro et al. [31] used JoinMap [36], the maps of Cavagnaro et al. [30], Just et al. [29] and Grzebelus et al. [32] used Mapmaker [37]. These two programs use very different mathematical and statistical procedures to construct the linkage groups [38] and these can account for the observed variation among carrot map sizes. Several linkage mapping studies have noted that marker distance and linkage group lengths were consistently larger with Mapmaker than JoinMap, regardless of the organism and the type of marker used [39–43]. This difference was observed even when both programs used the same mapping function [42, 43]. Because the multilocus-likelihood method used by Mapmaker assumes an absence of crossover interference while linkage maps constructed with JoinMap are more accurate and shorter when such interference is present [39, 42].

Another source of variation that may contribute to size differences observed among the carrot maps is the type of marker used. Uncorrected genotyping errors often detected with dominant AFLP markers, such as those predominantly used in earlier carrots maps with higher map size, can drastically increase the calculated map lengths [44, 45]. Such error is unlikely in our codominant SNP-based map. Marker scores across each linkage group were verified using CheckMatrix to eliminate miss-scored markers that may generate false double cross-overs (false double cross-overs can substantially increase linkage group length), therefore providing a solid support to the marker order.

The linkage map constructed in this study provides an unprecedented opportunity for integrating carrot genetic maps. Most of the SSR and gene-specific markers included in our map represent common data points across several linkage maps constructed for various carrot traits [18, 29–31] as well as chromosome anchoring markers [34]. In addition, over 300 SNPs mapped in this study are shared with a recently developed linkage map containing QTL for nematode resistance [46]. Integration of carrot genetic maps, including our dense SNP-based map, will rapidly increase marker density and include informative sequence-based markers relative to previously unsaturated map regions containing important traits. This will allow for straight forward identification of candidate genes to characterize traits with the upcoming carrot genome sequence [47].

High resolution linkage maps constructed with sequence-based markers are crucial for aiding in the assembly of next-generation sequence data used in genome sequencing projects. The map developed in this study, with 895 sequence-based markers and a resolution of 1.3 cM, is currently being used to facilitate the assembly of the carrot genome sequence.

### Genetics of anthocyanin pigmentation in carrot

**Figure 4:**
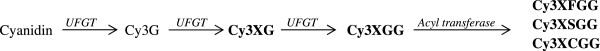
**Proposed anthocyanin biochemical pathway noting pigments and genes (in italics) involved in anthocyanin formation and compound modification in carrot roots.** In bold are anthocyanin pigments detected by HPLC in this and previous studies. Abbreviations correspond to the pigments full names, as in Table 1.

Based on examination of the structure of the five cyanidin glycosides present in carrot roots (Additional file 3), together with available information on anthocyanin biochemistry in carrot [48, 49] and other species [10, 50–53], a proposed scheme for cyanidin modification in carrot is presented in Figure 4. Based on this scheme, an inverse balance between the content of nonacylated Cy3XGG and acylated Cy3XCGG, Cy3XFGG, and Cy3XSGG is expected, with the balance modulated by the activity of an acyltransferase.

In the present study, the strong negative correlation found between the non acylated Cy3XGG and the total content of acylated anthocyanin forms (*r* = −0.99, P < 0.001) strongly suggests that acylation of Cy3XGG, presumably by an acyltransferase (in agreement with the proposed scheme in Figure 4), causes this shift from non-acylated to acylated anthocyanin forms. In addition, the co-localization of a large-effect QTL for Cy3XGG and two QTL for the major acylated anthocyanins (Cy3XSGG and Cy3XFGG) in a very narrow map region (3.6 cM) of chromosome 3, not only reinforces the notion that Cy3XGG is the chemical substrate for acylation, giving rise to Cy3XSGG and Cy3XFGG, but also suggests that this region is largely responsible for the genetic control of root anthocyanin acylation. Furthermore, our combined pigment and linkage analysis data for Cy3XGG provide evidence that suggests a single dominant gene controlling this trait. The clear bimodal distribution of Cy3XGG in the F_2_, a segregation ratio compatible with a simply inherited dominant trait, and the co-localization of this trait mapped both as a QTL and as single gene, all suggest that indeed a single dominant gene controls ‘high’ versus ‘low’ content of acylated anthocyanins in roots of 70349. We name this gene *Raa1*.

In addition to *Raa1*, two QTL with relatively smaller phenotypic effects conditioning anthocyanin acylation were detected in CH3, 23 cM apart from *Raa1*, and CH1, suggesting a complex regulation for modification of carrot pigments, with several genes probably influencing acylation.

Because acylation of carrot anthocyanins influences bioavailability [11, 20–22], an important property of dietary nutrients in human health, and it also influences pigment stability [19, 21, 23], important for the use of pigments in foods and as dyes, further characterization of *Raa1* is of interest. With the carrot genome sequence nearing completion [47], in combination with the sequence-based markers tightly linked to *Raa1* in the current 70349 genetic map, searching for candidate genes will be straight forward. A detailed characterization of *Raa1* may have a positive impact in carrot breeding programs aiming at developing carrots with higher nutraceutical value (e.g., with increased content of non-acylated anthocyanins) and also carrots with more stable pigments (i.e., with high proportion of acylated anthocyanins) for the food industry.

A previous study in carrot demonstrated that acylation of anthocyanins is important for transport into the vacuoles [54], suggesting that acylation may play an important role for the accumulation of stable pigments in carrot roots. However, in the present study, only a weak significant correlation (0.34) was found between purple root pigmentation (RTPE) and one of the acylated compounds (Cy3XSGG). In addition, *Raa1*, which conditions high versus low content of acylated anthocyanins, did not co-localize with either of the two RTPE QTL in the linkage map, suggesting that acylation does not have a major effect on the accumulation of root anthocyanins in the 70349 genetic background.

### Mapping of anthocyanin QTL

The study presented here represents the first comprehensive QTL analysis of anthocyanin pigmentation and pigment modification performed in carrot. Using quantitative data we identified 15 QTL covering eight genomic regions associated with accumulation of four cyanidin derivatives and with the percentage of purple pigmentation (RTPE) in carrot root. Consistent with the two-gene model observed for root purple color segregation in F2-F4 families, two QTL on chromosomes 3 and 1 were detected to account for variation in RTPE.

With the exception of the Cy3XGG QTL in the 27.0-27.7 cM region of CH3, the QTL with the largest phenotypic effect for all the anthocyanin glycosides and for root total pigment content, which explained 36.6 - 59.2% of the variation, were associated with the 43.1-50.1 cM region of CH3 and best associated with marker K0627. We propose that this QTL region includes a gene that controls the primary regulatory mechanism underlying anthocyanin accumulation. Detection of ten additional QTL covering six genomic regions associated with the accumulation of individual anthocyanin compounds, located on chromosome 2, 3, 4, 6 and 8 indicates the complexity of the genetic control of anthocyanin accumulation in carrot. Based on studies in other crops these QTL could be involved in anthocyanin biosynthesis, accumulation, and chemical modification (e.g., glycosylation, acylation) necessary for the stable storage of these pigments, as has been reported for anthocyanin accumulation and diversification in other species. For example, in raspberry, Bushakra et al. [55] detected 24 QTL associated with the accumulation and diversity of different cyanidin derivatives, while in grapes, Fournier-Level et al. [56] detected multiple QTL associated with cyanidin methylation and accumulation.

### Synteny and putative orthologous anthocyanin loci among carrot maps

Several carrot maps for various traits of interest, including anthocyanin and carotenoid pigmentation, have been developed [18, 26, 29, 30] but common markers across linkage maps carrying similar traits in different genetic backgrounds were usually lacking in these previous studies.

In the present study, the use of sequence-based markers for chromosome 3 in common across three linkage maps of carrot harboring root and petiole anthocyanin traits revealed high synteny among the maps (Figure 3). This, in combination with the use of markers tightly linked to the anthocyanin mapped traits, revealed that the genetic factors conditioning root pigmentation in populations 70349 (QTL-RTPE) and 2170 (P3) are different from P1, previously reported in 10117 [18]. The syntenic correspondence between RTPE (in 70349) and *P*_*3*_ (in 2170) and the tight linkage of both traits to marker K0627 in both maps suggests that this region controls root anthocyanin accumulation in both genetic backgrounds.

Purple carrots played an important role in the domestication of carrot as a tap root crop [63]. Based upon historical documents, it is believed that the first domesticated carrot roots were purple and yellow and such domestication occurred in Central Asia, with orange carrots representing a secondary and European domestication event [63]. Our comparative map analysis for anthocyanin accumulation in carrot represents not only a breeding tool but also provides further insight into carrot domestication. The fact that two different genes control anthocyanin root pigmentation in unrelated genetic backgrounds suggests that independent mutations and human selection events may have contributed to the domestication of purple carrots. Petiole anthocyanin pigmentation segregated as a simply-inherited trait in populations 70349 and 2170, and was mapped for the first time. Furthermore, in this study, we present clear evidence for pleiotropy at a single gene controlling both petiole and root pigmentation in these two populations. This is in contrast to a previous study which found, in a population not included in this study, that root pigmentation and petiole pigmentation were controlled by different genes [17]. Coincidently with our results in populations 70349 and 2170, a single gene, *AN1*, has been found to condition anthocyanin pigmentation in different tissues (tuber skin and foliage) of potato [63–65]. Together, these findings emphasize the observation that anthocyanin biosynthesis, accumulation and structure is a complex trait in carrot, as has been observed in several other plant species. Even beyond identifying candidate genes for QTL identified in this study, more genes conditioning this trait are likely to be discovered elsewhere in the carrot genome.

Despite the high synteny observed among the three maps, their total length varied considerably. While the map lengths were comparable between population 2170 (49.0 cM) and population 10117 (55.5 cM), the former maps were shorter than the 70349 map of CH3 (86.1 cM). This is likely due to the fewer number of markers mapped in 2170 and 10117 compared to 70349, with the former maps lacking markers in one (10117) or both (2170) of the terminal chromosome ends, as is evident from map comparisons in figure 3. The mapping of these eight sequence-based and common markers allowed for a close comparison of these 3 maps, and will contribute to map integration while increasing marker informativeness and marker saturation in the 2170 and 10117 backgrounds. More importantly, this will result in an immediate increase in marker saturation in nearby regions of P1, P3 and Raa1, which will facilitate tracking these genes in breeding programs, and will further facilitate searching for candidate genes in the upcoming carrot genome sequence.

## Conclusions

An informative high resolution SNP-based genetic map of carrot was constructed and used for detection and mapping of root anthocyanin QTL. In population 70349, the mapping of 15 QTL across 6 carrot chromosomes suggests a more complex regulation for this trait than previously described in a previously unstudied genetic background, with many genes probably influencing pigment biosynthesis, compound modification and/or accumulation. Of particular interest, a 12 cM region of chromosome 3 harboring co-localized QTL with largest phenotypic effect for root total pigment content (RTPE) and four anthocyanin glycosides, as well as the simply-inherited gene *P*_*3*_, which was largely responsible for the genetic control of anthocyanin pigmentation in both roots and petioles. In addition, quantitative data analysis for each anthocyanin compound provided the opportunity to identify a large effect QTL and a simply-inherited gene, *Raa1*, that was also on chromosome 3 and associated with anthocyanin acylation. Comparative map analysis revealed that neither of these chromosome regions that were associated with anthocyanin pigmentation and acylation, respectively, corresponded to the *P*_*1*_ gene initially reported for root purple pigmentation.

The present study increases the current knowledge on the genetic control for this complex trait in carrot. These data, together with the molecular resources generated herein (e.g. the high-resolution SNP map and the development of markers in common across different linkage maps) and the upcoming carrot genome sequence will facilitate cloning of the genetic factors controlling both anthocyanin accumulation and acylation. Characterization of such genes will have a positive impact on carrot breeding programs aiming at increasing pigment concentration and anthocyanin profiles, depending on the end-market use.

## Methods

### Plant material

Inheritance of purple pigmentation was studied in F_2_, F_3_ and F_4_ families derived from an initial cross between P4201 and B6320. P4201 is an inbred line with purple outer phloem and yellow xylem storage roots and purple leaves that was derived from a cross between inbred P9547 (Figure 1), with purple xylem and phloem root color derived from Central Anatolia, and B2566, an inbred with orange root color from diverse European sources. B6320 is an inbred with orange roots and green petioles derived from the European open-pollinated cultivars Nantes and Camberly. A single F_1_ plant with purple root outer phloem and yellow xylem, and purple leaves, was self-pollinated to produce the F_2_ population 70349 (*N* = 519), which was used for genetic mapping studies. For phenotyping, plants of the F_2_ population were grown in pots under greenhouse conditions in 2007. After phenotyping as described below, roots were vernalized at 1–2°C, planted at the University of Wisconsin West Madison Agricultural Research Station (WMARS), and individual plants were self-pollinated to produce F_3_ families. A subset of the F_2_ and the F_3_ families were field-grown at El Centro, California, in 2009, and phenotyped for root pigmentation. Individual F_3_ plants underwent another cycle of self-pollination and their F_4_ progenies were grown in Madison-WI and phenotyped in 2012.

For comparison purposes, linkage analyses were also performed in two mapping populations unrelated to population 70349 but also segregating for root and petiole purple pigmentation. Population 2170 was an F_3_ family (N = 65) derived from a cross between a purple rooted carrot with purple leaves derived from an intercross between PI652188 (a purple carrot from China, and the ultimate purple source in 2170) and PI326011, an orange-rooted European carrot with green leaves. An F_2_ plant of this family with the same phenotype as the purple parent was self-pollinated to generate the F_3_ family evaluated. The other mapping population, 10117, was an F_2_ family (N = 72) derived from a cross between B1896 (a true-breeding inbred with yellow roots derived from a cross between PI173687, a population from eastern Turkey segregating for presence or absence of purple root color, and B493, an inbred with orange roots and green petioles derived from diverse European sources) and B7262 (Figure 1), an inbred with purple outer phloem and orange inner phloem and xylem, and purple leaves from the same cross as B1896). This population was previously characterized in more detail by Yildiz et al. [18]. Thus, the ultimate genetic source of purple color in the three mapping populations in this study varied geographically and phenotypically.

### Phenotyping and segregation analysis

Phenotyping purple color in 70349 and its F_3_ - F_4_ derivatives was performed on the basis of the presence or absence of purple pigmentation in carrot roots as described by Simon [17]. In addition, the percentage of purple pigmentation covering the root surface, which estimates the root total pigment content in the 70349 background, was scored visually and recorded in 70349 (from here on referred to as root “RTPE” for “root total pigment estimate”). Root tissues from purple carrots were sampled for anthocyanin HPLC analysis. Purple pigmentation in leaves of 70349 was evaluated on the basis of presence or absence of purple color in the leaf petioles, as described previously [17]. Phenotyping was done on a presence/absence basis as in this previous study for anthocyanin pigmentation in roots and petioles of population 2170 and roots of population 10117. Data from the F_2_, F_3_ and F_4_ carrot segregating populations were analyzed using the χ^2^ method to test the ratios expected for each character.

### HLPC analysis

Tissue samples containing visible purple pigmentation were obtained from roots of the 70349 mapping population. The samples were lyophilized and anthocyanins were extracted with acidified methanol, followed by high-performance liquid chromatography (HPLC) analysis of anthocyanin pigments as described by Kurilich et al. [11]. Table 1 lists the five major carrot anthocyanin pigments (all cyanidin derivatives) identified and quantified in the present study. The data were expressed as percentage concentration of a given pigment relative to the total anthocyanin content, which derived from the sum of the content of the five individual anthocyanin compounds. Pairwise Spearman rank correlation values among anthocyanin pigments and traits were obtained using SAS v.9.1 [57].

### Construction of the framework linkage map in 70349

Total genomic DNA of individual plants from 70349, 2170 and 10117 populations was isolated from lyophilized leaves following the protocol described by Murray and Thompson [58] and quantified using Pico Green (Invitrogen, Paisley, UK).

The linkage map was constructed using 187 F_2_ individuals from 70349. A collection of 4000 published SNPs developed from carrot transcriptome data [35] and 40 published SSR markers with known chromosome location [30] were used. SNPs were genotyped using the KASPar chemistry, which is a competitive allele-specific PCR SNP genotyping system using FRET quencher cassette oligos (http://www.lgcgroup.com). SNPs were coded with a ‘K’ followed by four digit numbers (e.g. K0001). SNPs located within carotenoid or anthocyanin biosynthetic genes were labeled according to the gene abbreviation. SSR primer pairs were evaluated using a fluorescent method as described before [59].

JoinMap 4.0 software [36] was used for mapping. Scores of all markers used for mapping were converted into genotype codes using the A/H/B system for co-dominant and A/C, B/D system for dominant markers segregating in F_2_ population. The linkage groups (LGs) were obtained at a LOD threshold value >3.0. Regression mapping algorithm and Haldane’s mapping function was used to calculate genetic distances among marker loci. Markers and genotypes with more than 10% of missing data were excluded from the analysis. The degree of marker segregation distortion in the F_2_ was determined by marker data comparison against the expected 3:1 and 1:2:1 ratio for dominant and codominant markers, respectively, using Chi square tests, where significant distortion was declared at P < 0.01 [60]. The marker order in each linkage group was examined for inconsistencies leading to false double recombination events using CheckMatrix (http://www.atgc.org/XLinkage). Markers with more than one inconsistent score were removed. In order to reduce the complexity of the final map figure, redundant markers, considering as such those that had no recombination among them and therefore shared the same map position, were removed from the image, leaving a single marker -the marker with the least amount of missing data- per map position. A complete list with all the mapped markers, including redundant markers, is provided in Additional file 6.

Map positions of SSR markers and SNP markers corresponding to anthocyanin and carotenoid biosynthetic genes with known chromosome location [18, 30] were used to anchor the linkage groups to carrot chromosomes. After chromosome assignment, LGs were oriented and numbered according to the chromosome orientation and classification of Iovene et al. [34].

### QTL analysis

QTL analysis was performed using R/qtl with the multiple imputations method [61]. QTL detection included preliminary QTL identification using scanone followed by QTL modeling. Briefly, the QTL with the largest LOD value from the ‘scanone’ analysis was added to the QTL model and if the model was significant, the QTL was retained. This process was then repeated using ‘addqtl’ instead of ‘scanone’, followed by QTL modeling and testing for interactions among the QTL, until adding additional QTL to the model was no longer significant. The support intervals for the map locations of QTL were calculated using a 1.5 LOD drop interval which is considered the best approximation to a 95% support interval in QTL mapping.

### Comparative mapping of anthocyanin traits across diverse carrot genetic backgrounds

In addition to the 70349 mapping population, the phenotypic traits controlling root and petiole purple pigmentation, and several common markers (SNPs, SSRs) and genes (*FLS*, *F3H*, *PSY2*) were mapped in F_2_ populations 10117 and 2170, which were unrelated to 70349 (see subsection ‘plant material’). The anthocyanin genes *F3H* (flavanone 3-hydroxylase) and *FLS* (flavanone 3'hydroxylase), and the carotenoid gene *PSY2* (phytoene synthase-2), as well as the SSRs used, were known to locate in chromosome 3 based upon results from previous linkage studies in carrot [18, 29].

Primer pairs for 6 SNPs selected from CH3 of 70349, as well as for *FLS*, *F3H* and *PSY2* were designed using Primer3 software [62] and used in polymerase chain reactions (PCR) of 20 μl final volume containing 1X DNA polymerase buffer, 0.02 mM of each dNTP, 0.25 μM of each primer, 0.2 μl Taq polymerase (MBI, Fermentas, USA) and ~50 ng of genomic DNA. PCR conditions were: initial denaturation at 94°C for 2 min, followed by 25 cycles of 94°C for 30 sec, appropriate annealing temperature for 30 sec, and 72°C for 45 sec, and a final step at 72°C for 10 min. Sequencing reactions were performed in 5 μl final volume consisting of 1.75 μl of water, 1 μl of 5 μM primer, 0.75 μl of 5 × BigDye®3.1 sequencing buffer, 0.5 μl of Big-Dye®3.1 and 1 μl of PCR product, previously diluted 1:10 with water. Big dye reaction conditions were 25 cycles of 96°C for 10 sec, and 58°C for 2 min, and a final step at 72°C for 5.0 min. Sequencing was performed at the University of Wisconsin Biotechnology Center, and sequence analysis used Sequencher software version 4.8 (GeneCodes Corporation, Ann Arbor, MI). SSR markers were evaluated using a fluorescent method as described by Iorizzo et al. [59]. Phenotyping and mapping of purple pigmentation in roots and leaves of 2170, and in roots of 10117, was described above.

## Electronic supplementary material

Additional file 1: **Phenotypic variation for root total pigment content (RTPE) in 70349.** (PDF 91 KB)

Additional file 2: **Frequency distribution of ‘root total pigment content’ (RTPE) and of individual anthocyanin pigment compounds in roots of 70349.** (PDF 42 KB)

Additional file 3: **Molecular structure of anthocyanins from carrot root.** (PDF 198 KB)

Additional file 4: Figure S1: Pigment distribution and correlation analysis among cyanidin derivatives and ‘root total pigment content’ (RTPE) in 70349 population. **Table S2.** Pair-wise Spearman rank correlation values among root anthocyanin pigments in 70349. (PDF 262 KB)

Additional file 5: **Marker genotype analysis in individuals of 70349 mapping population displayed vertically across the nine carrot chromosomes using Checkmatrix.** (PDF 2 MB)

Additional file 6: **Complete list of mapped markers per chromosome.** (XLSX 40 KB)

Additional file 7: **Allele interaction analysis for closest markers associated with QTL for anthocyanin compounds and root total pigmentation (RTPE).** (XLSX 154 KB)

## Abbreviations

CHs: Chromosomes
Cy3XGG: Cy-3-(2”-xylose-6-glucose-galactoside)
Cy3XG: Cy-3-(2”-xylose-galactoside)
Cy3XSGG: Cy-3-(2”-xylose-6”-sinapoyl-glucose-galactoside)
Cy3XFGG: Cy-3-(2”-xylose-6”-feruloyl-glucose-galactoside
Cy3XCGG: Cy-3-(2”-xylose-6”-(4-coumuroyl)glucose-galactoside)
DArT: Diversity arrays technology
HPLC: High-performance liquid chromatography
QTL: Quantitative trait loci
RT: Retention time
RTPE: Root total pigment estimate
SNP: Single nucleotide polymorphism
SSR: Simple sequence repeat.
