# Supplementary material for: A gene-derived SNP-based high resolution linkage map of carrot including the location of QTL conditioning root and leaf anthocyanin pigmentation
Source: BMC Genomics. 2014 Dec 16;15(1):1118. doi: 10.1186/1471-2164-15-1118 (PMC4378384; doi:10.1186/1471-2164-15-1118)
Supplement: Supplementary file 1 — Additional file 1: Phenotypic variation for root total pigment content (RTPE) in 70349. (PDF 91 KB) [file 12864_2014_6833_MOESM1_ESM.pdf]

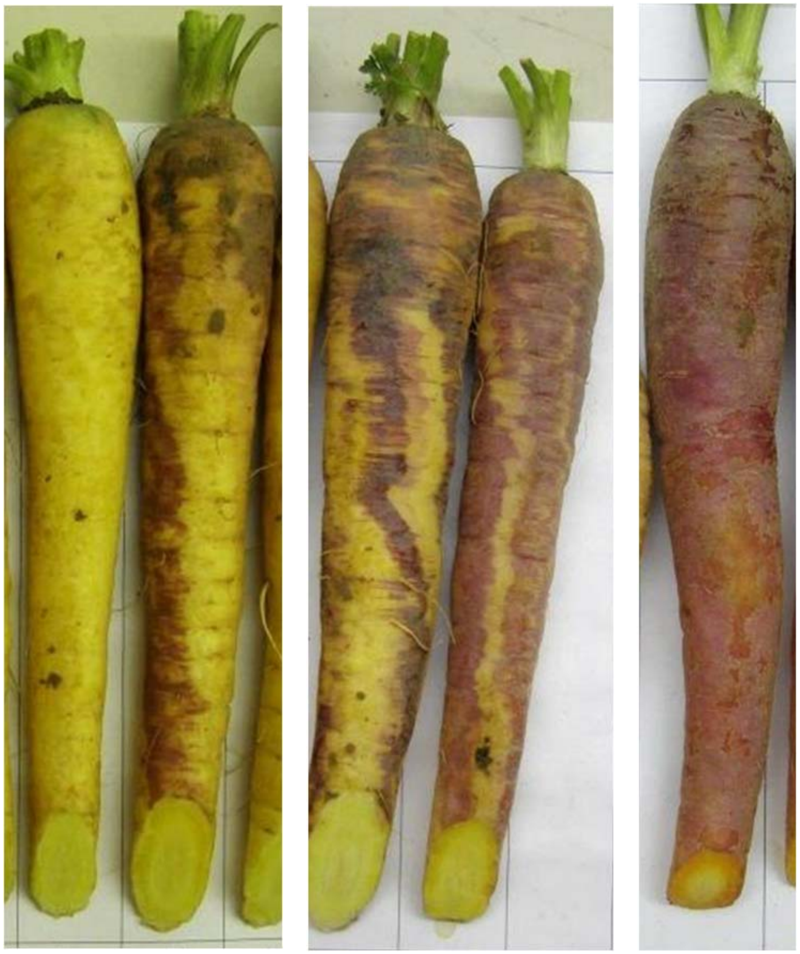

Additional file 2. Examples of variation observed for root total pigment estimate (RTPE) in the 70349 population ranging from 0% to 20%, 30%, 80%, and 100% (left to right)
