# Supplementary material for: A gene-derived SNP-based high resolution linkage map of carrot including the location of QTL conditioning root and leaf anthocyanin pigmentation
Source: BMC Genomics. 2014 Dec 16;15(1):1118. doi: 10.1186/1471-2164-15-1118 (PMC4378384; doi:10.1186/1471-2164-15-1118)
Supplement: Supplementary file 2 — Additional file 2: Frequency distribution of ‘root total pigment content’ (RTPE) and of individual anthocyanin pigment compounds in roots of 70349. (PDF 42 KB) [file 12864_2014_6833_MOESM2_ESM.pdf]

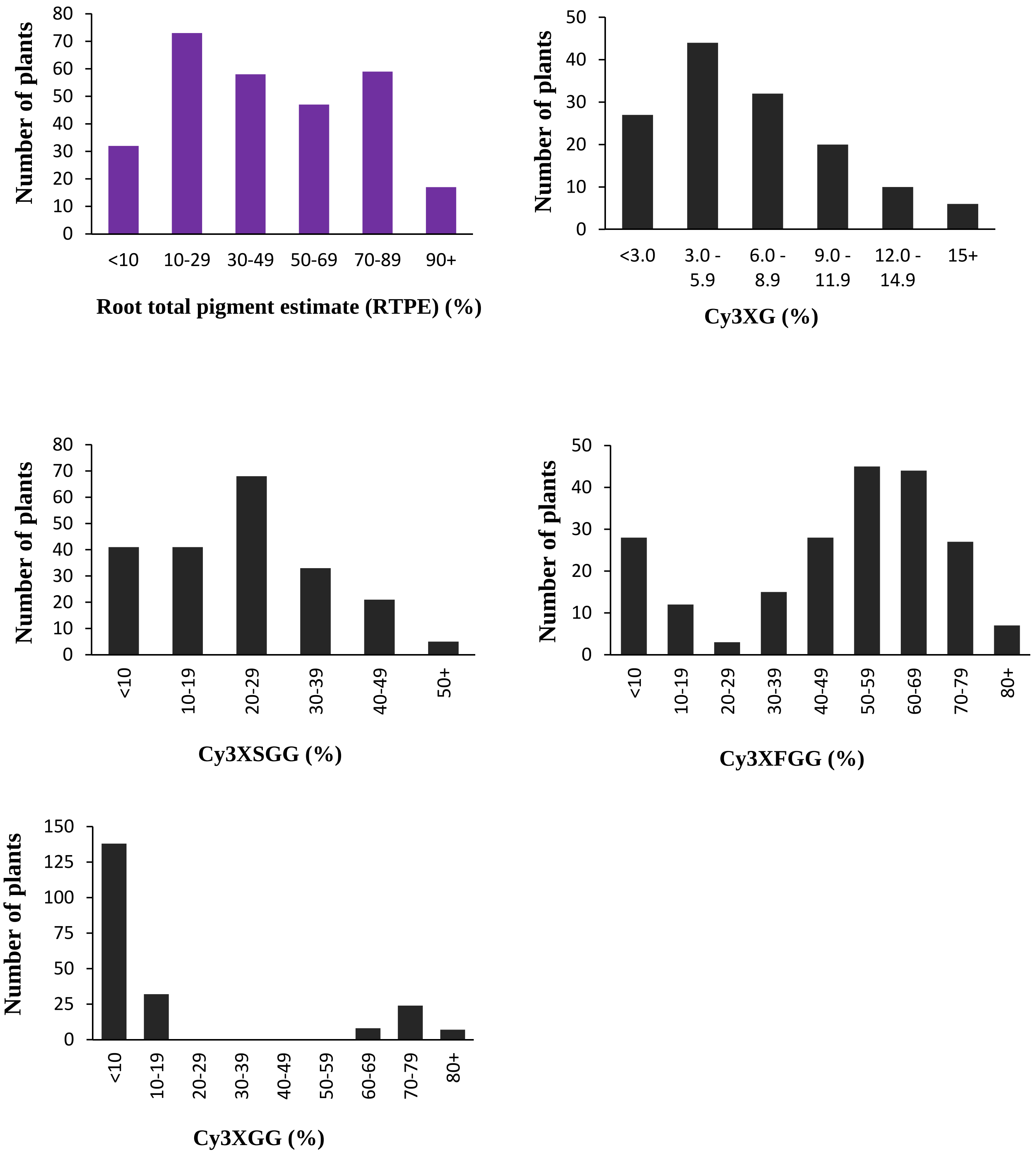

Additional file 3. Frequency distribution of 'root total pigment estimate' (RTPE) and of individual anthocyanin pigment compounds in roots of 70349. Pigment Cy3XCGG was not included because it was only detected in trace amounts in this population. Distribution of RTPE was based on data from 279 F_2 individuals, whereas analysis of pigment compounds used HPLC data from 208 plants. In both cases, only roots containing visible purple pigmentation were used.
