# Supplementary figures and images for: A gene-derived SNP-based high resolution linkage map of carrot including the location of QTL conditioning root and leaf anthocyanin pigmentation
Source: BMC Genomics. 2014 Dec 16;15(1):1118. doi: 10.1186/1471-2164-15-1118 (PMC4378384; doi:10.1186/1471-2164-15-1118)

## Additional file 7: Molecular structure of anthocyanins from carrot root

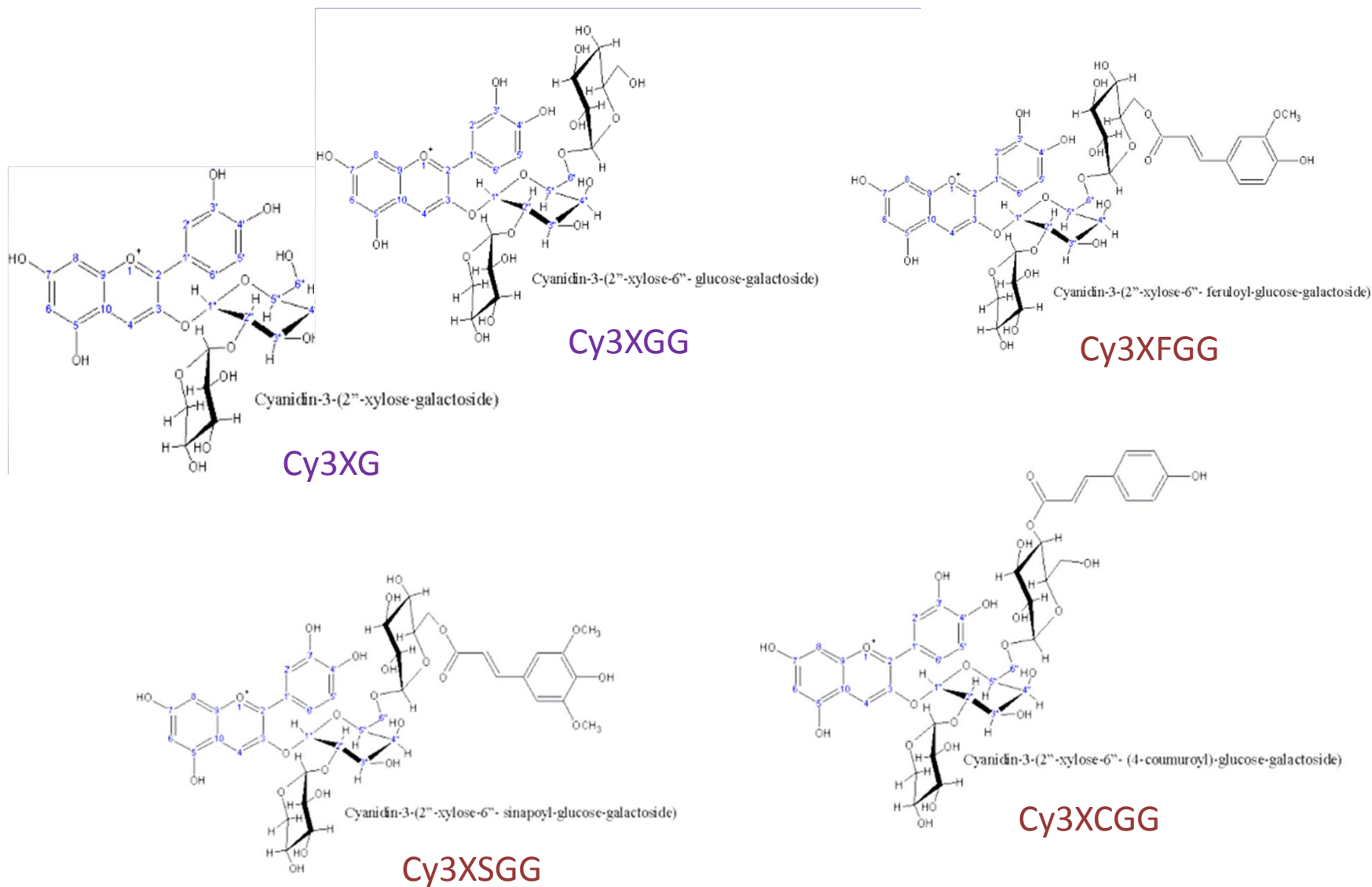

Supplement: Supplementary file 3 — Additional file 3: Molecular structure of anthocyanins from carrot root. (PDF 198 KB) [file 12864_2014_6833_MOESM3_ESM.pdf]
