# Supplementary material for: A gene-derived SNP-based high resolution linkage map of carrot including the location of QTL conditioning root and leaf anthocyanin pigmentation
Source: BMC Genomics. 2014 Dec 16;15(1):1118. doi: 10.1186/1471-2164-15-1118 (PMC4378384; doi:10.1186/1471-2164-15-1118)
Supplement: Supplementary file 4 — Additional file 4: Figure S1: Pigment distribution and correlation analysis among cyanidin derivatives and ‘root total pigment content’ (RTPE) in 70349 population. Table S2. Pair-wise Spearman rank correlation values among root anthocyanin pigments in 70349. (PDF 262 KB) [file 12864_2014_6833_MOESM4_ESM.pdf]

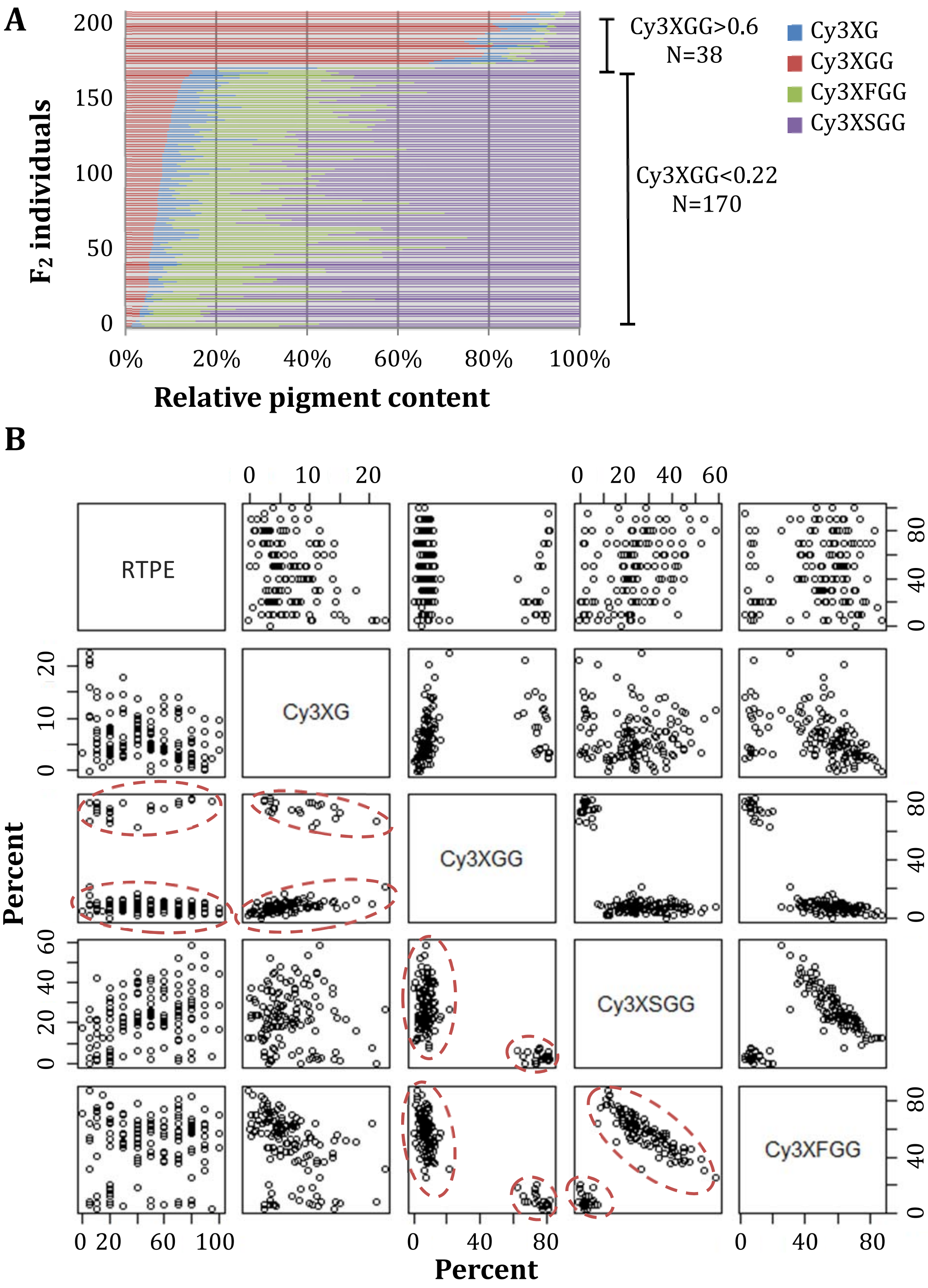

Additional file 4: figure S1. Pigment distribution and correlation analysis among cyanidin derivatives Cy3XG, Cy3XGG, Cy3XSGG and Cy3XFGG, and 'root total pigment estimate' (RTPE) in 70349 population. Only plants with root purple pigmentation (N=208) were evaluated by HPLC anthocyanin analysis. **A.** Relative content (%) of four cyanidin derivatives in 208 purple-rooted individuals of 70349. **B.** Scatter plot for pair-wise correlations among anthocyanin pigments content (%) and RTPE. Circles indicate individuals with Cy3XGG < 22% and Cy3XGG > 60%.

Additional file 4: table S2. Pair-wise Spearman rank correlation values among root anthocyanin pigments in 70349

	**RTPE**	**Cy3XG**	**Cy3XFGG >0.2**	**Cy3XFGG <0.2**	**Cy3XSGG >0.1**	**Cy3XSGG <0.1**
**Cy3XG**	-0.24					
**Cy3XSGG**	**0.34**	0.01				
**Cy3XFGG**	0.005	**-0.54**				
**Cy3XGG<0.2**	-0.23	**0.65**	**-0.48**		0.12	
**Cy3XGG>0.6**	0.44	-0.47		**-0.66**		-0.22
**Cy3XFGG>0.2**					**-0.87**	
**Cy3XFGG<0.2**						0.14

Significant correlations (P<0.001) are indicated in bold.
