# Supplementary material for: A gene-derived SNP-based high resolution linkage map of carrot including the location of QTL conditioning root and leaf anthocyanin pigmentation
Source: BMC Genomics. 2014 Dec 16;15(1):1118. doi: 10.1186/1471-2164-15-1118 (PMC4378384; doi:10.1186/1471-2164-15-1118)
Supplement: Supplementary file 5 — Additional file 5: Marker genotype analysis in individuals of 70349 mapping population displayed vertically across the nine carrot chromosomes using Checkmatrix. (PDF 2 MB) [file 12864_2014_6833_MOESM5_ESM.pdf]

**Additional file 5. Marker genotype analysis in individuals of 70349 mapping population displayed vertically across the nine carrot linkage groups using Checkmatrix.** The full set of mapped markers (N= 894) was analyzed here. The genotypes are arranged along the x-axis and the loci are displayed in linear order along the y-axis. Red indicate parent A allele; blue indicate parent B allele; yellow indicated heterozygote loci; gray indicate missing data. For each chromosome and from right to left; the linkage group (LG) is represented graphically (with genetic distances on the right), followed by a column indicating the number of inconsistent scores (whenever present) for a given map position of the LG, followed by column listing the marker's ID. The horizontal graph in the lower part of the image shows the relative proportion of the two parental haplotypes and the number of double crossovers for each individual.

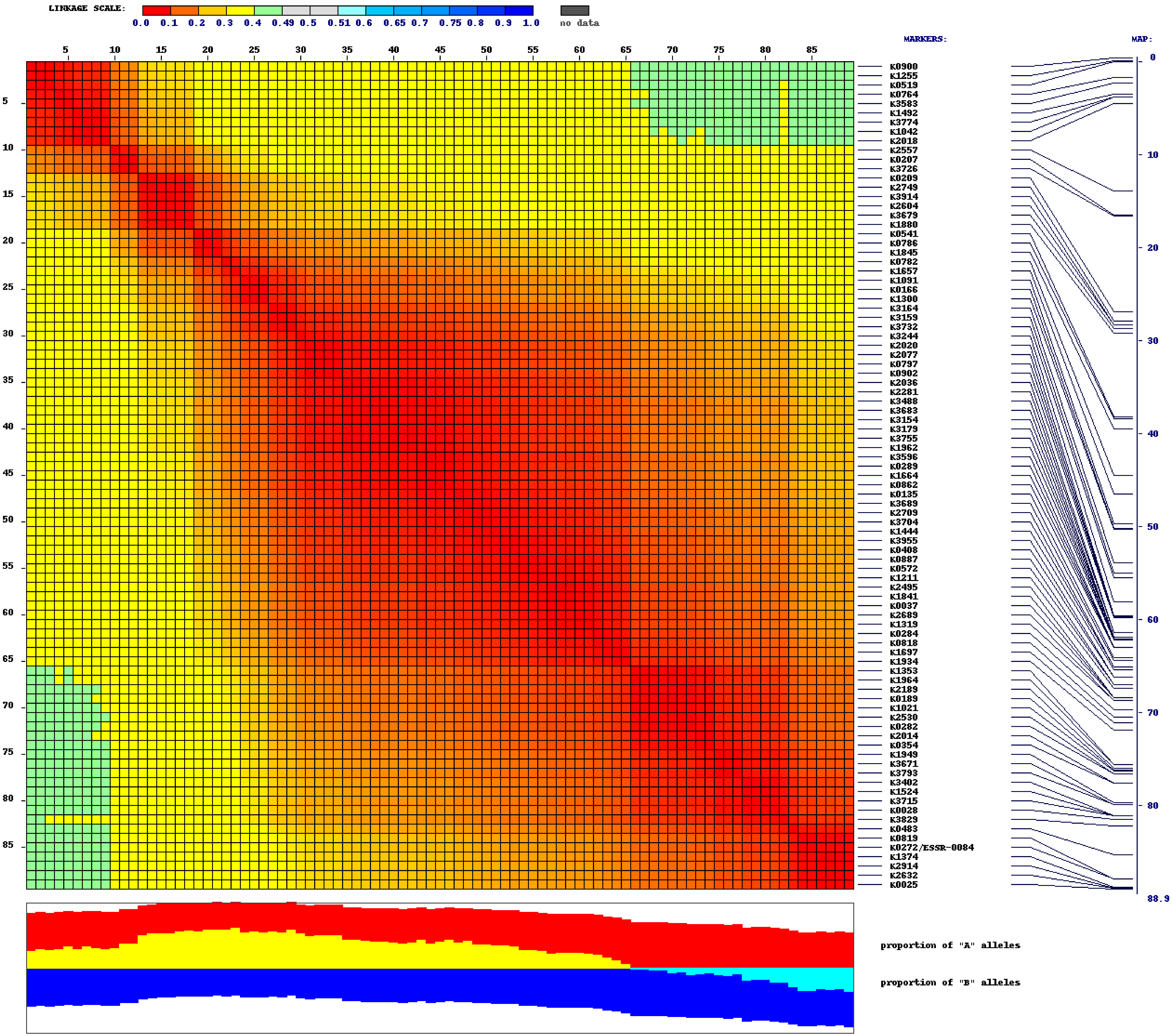


MAP: 05.resultsdir/full_correction.1.map MATRIX: 05.resultsdir/full_correction.1.pairs_ CUTOFF: 0.4

# Chromosome 1 - Page 3

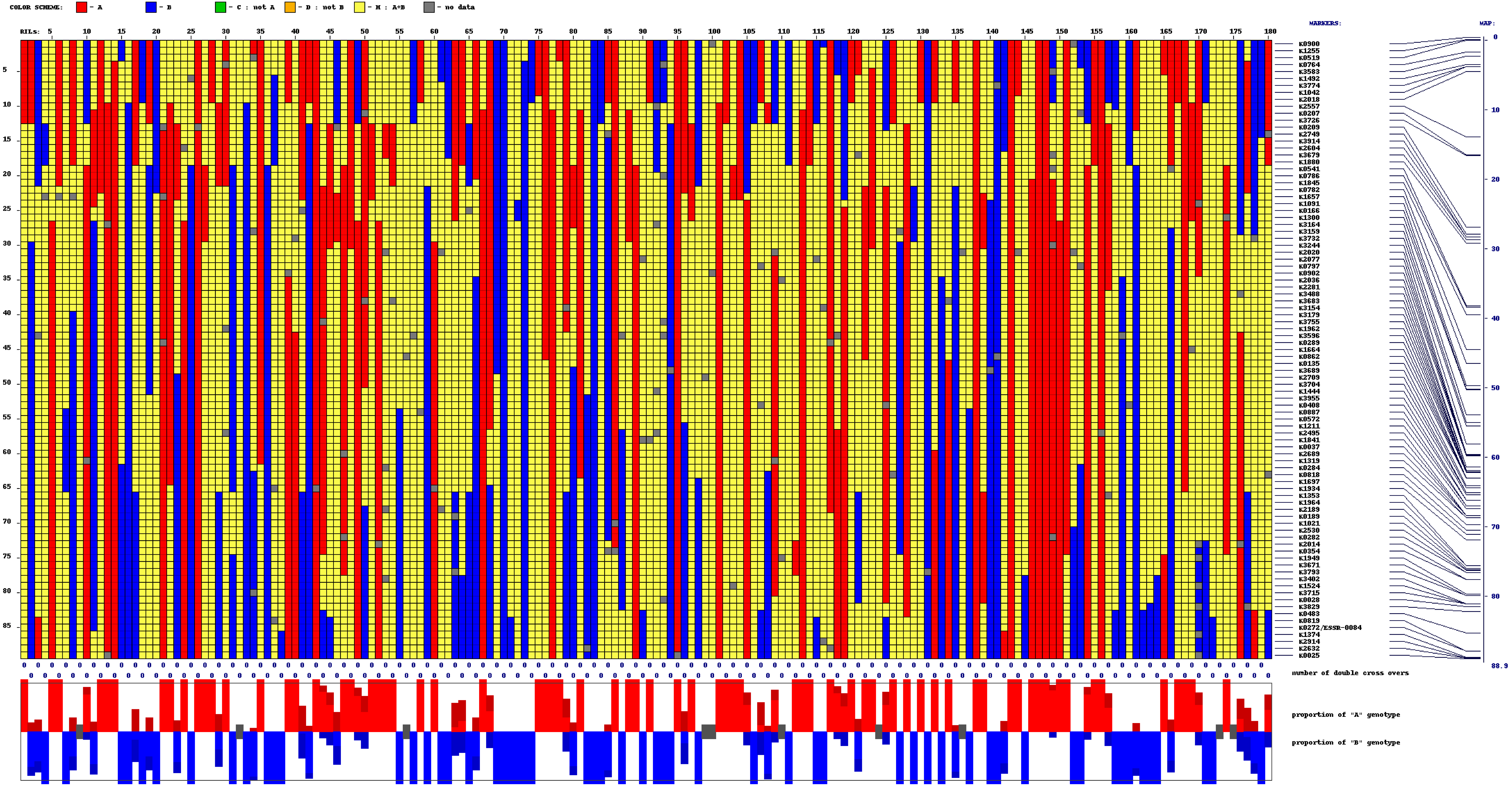


MAP: 05.resultsdir/full_correction.1.map LOCUS FILE: 05.resultsdir/05.full_correction.1.inputmatrix

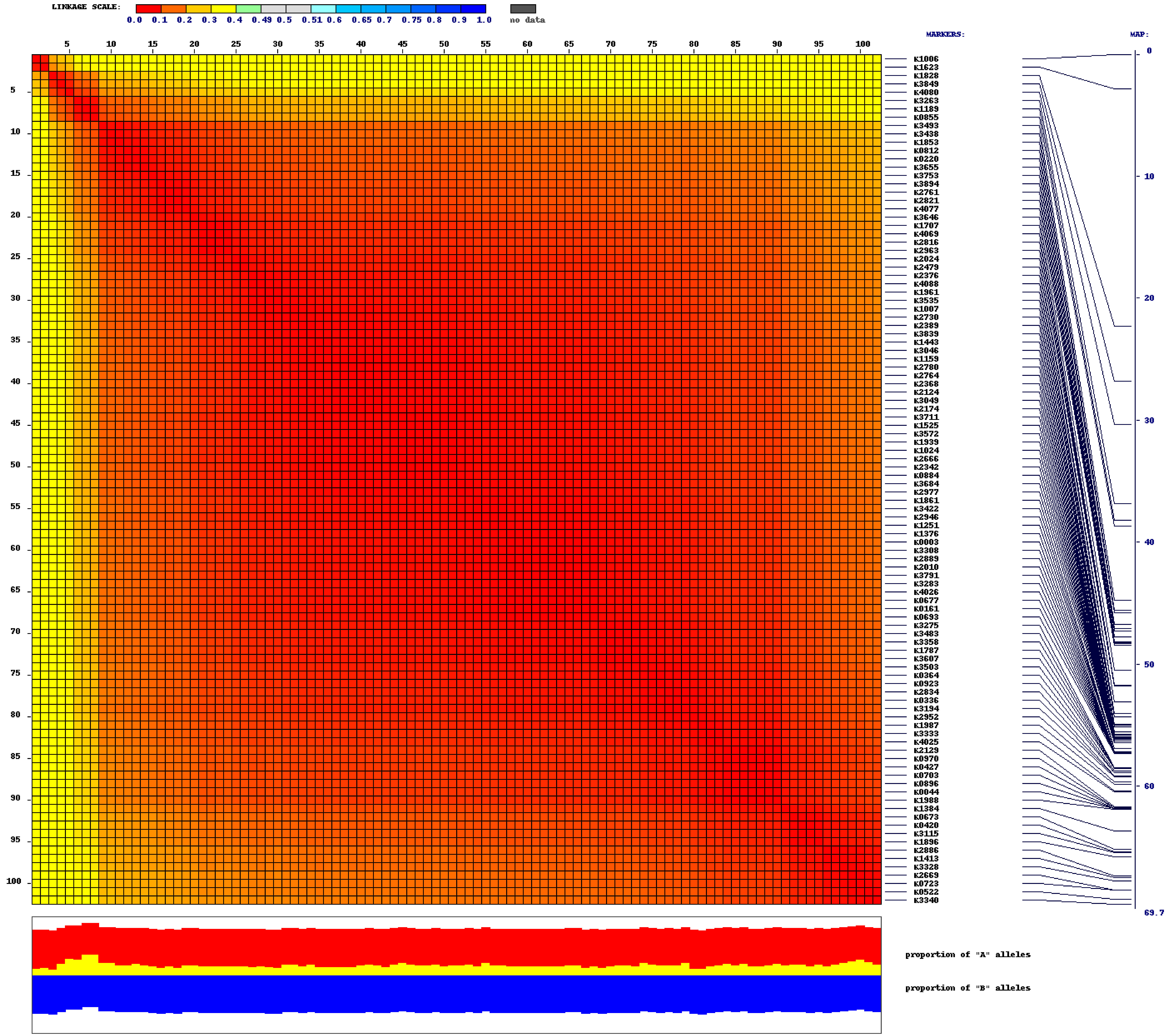


MAP: 05.resultsdir/full_correction.2.map MATRIX: 05.resultsdir/full_correction.2.pairs CUTOFF: 0.4

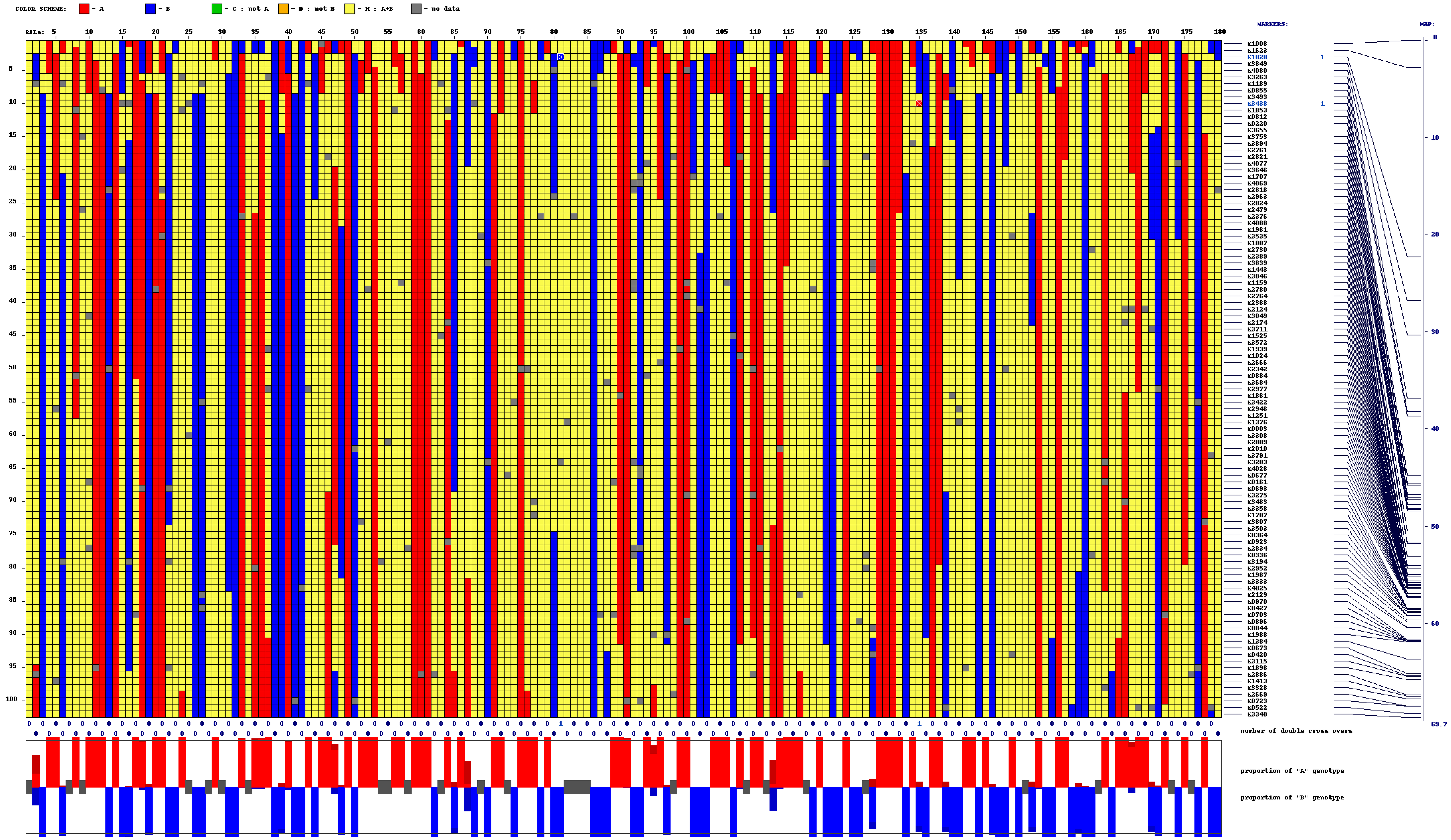


MAP: 05.resultsdir/full_correction.2.map LOCUS FILE: 05.resultsdir/05.full_correction.2.inputmatrix

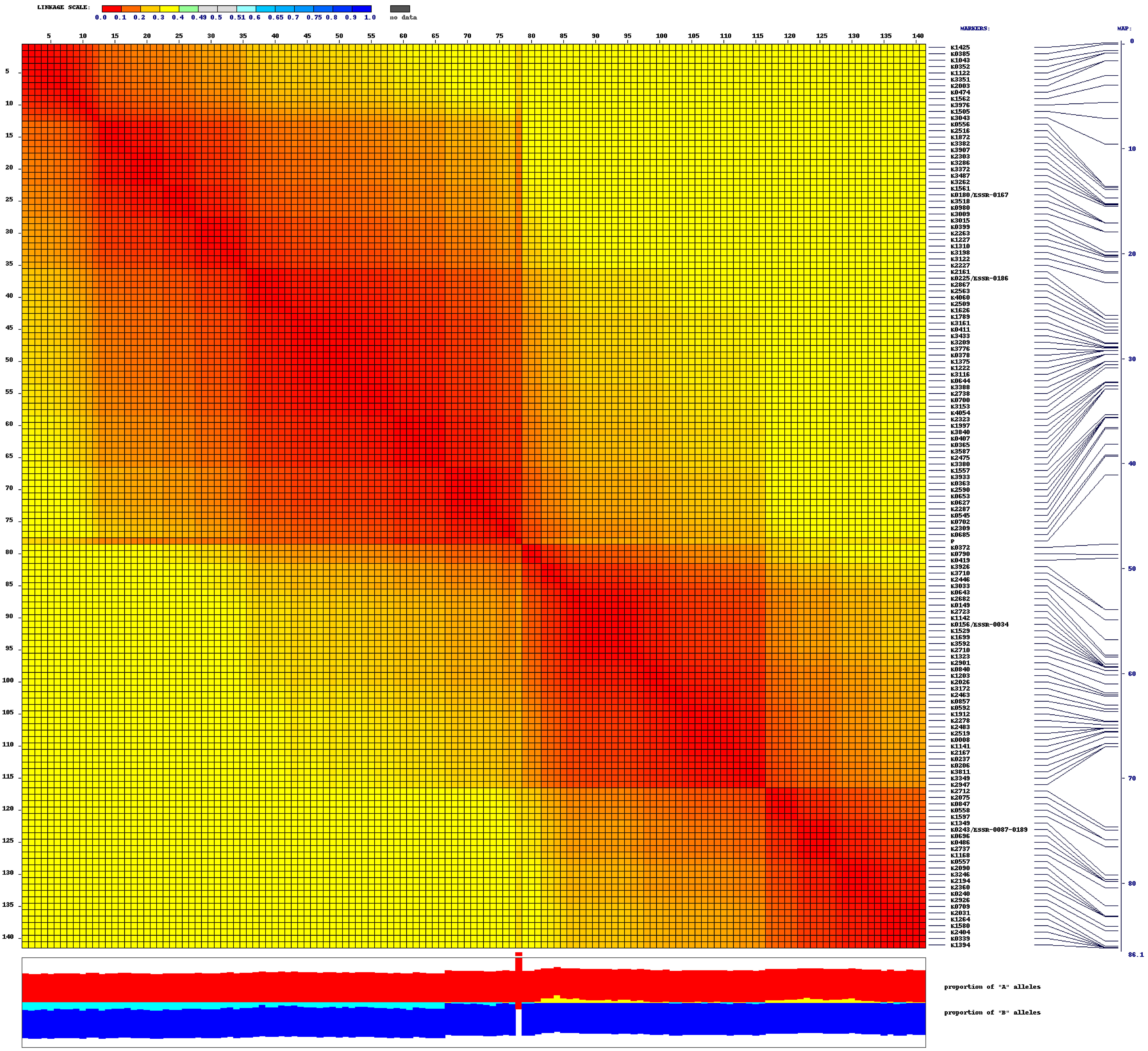


MAP: 05.resultsdir/full_correction.3.map MATRIX: 05.resultsdir/full_correction.3.pairs_all CUTOFF: 0.4

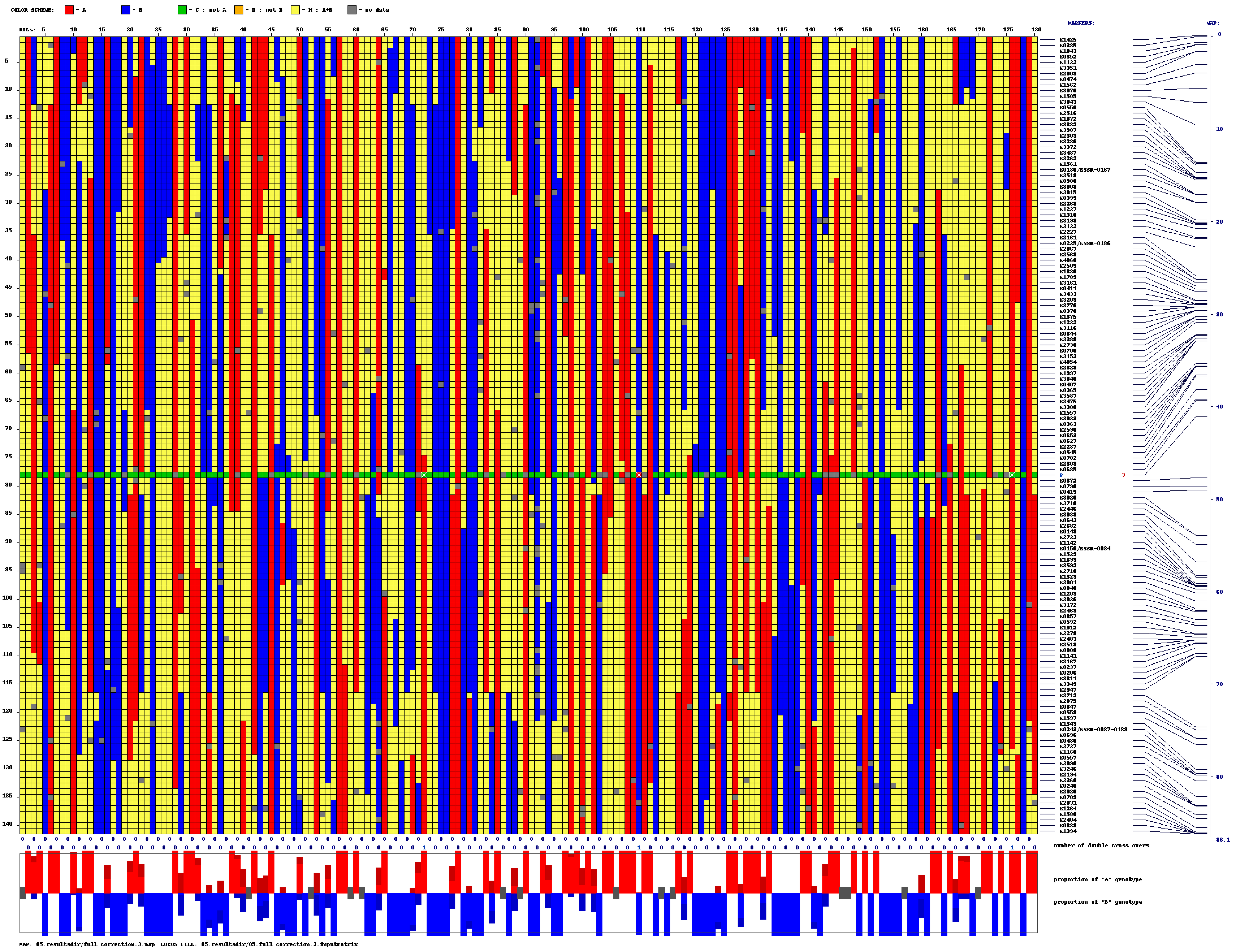
COLOR SCHEME: - A - B - C : not A - D : not B - H : A+B - no data
RILs:
MARKERS:
MAP:
K1425
K0385
K1043
K0352
K1122
K3351
K2003
K0474
K1562
K3976
K1505
K3043
K0556
K2516
K1872
K3382
K3907
K2303
K3286
K3372
K3487
K3262
K1561
K0180/ESSR-0167
K3518
K0980
K3009
K3015
K0399
K2263
K1227
K1310
K3198
K3122
K2227
K2161
K0225/ESSR-0186
K2867
K2563
K4060
K2509
K1626
K1789
K3161
K0411
K3433
K3209
K3776
K0378
K1375
K1222
K3116
K0644
K3388
K2738
K0700
K3153
K4054
K2323
K1997
K3840
K0407
K0365
K3587
K2475
K3380
K1557
K3933
K0363
K2590
K0653
K0627
K2287
K0545
K0702
K2309
K0685
P 3
K0372
K0790
K0419
K3926
K3710
K2446
K3033
K0643
K2682
K0149
K2723
K1142
K0156/ESSR-0034
K1529
K1699
K3592
K2710
K1323
K2901
K0840
K1203
K2026
K3172
K2463
K0857
K0592
K1912
K2278
K2483
K2519
K0008
K1141
K2167
K0237
K0206
K3811
K3349
K2947
K2712
K2075
K0847
K0558
K1597
K1349
K0243/ESSR-0087-0189
K0696
K0486
K2737
K1168
K0557
K2090
K3246
K2194
K2360
K0240
K2926
K0709
K2031
K1264
K1580
K2404
K0339
K1394
86.1
number of double cross overs
proportion of "A" genotype
proportion of "B" genotype
MAP: 05.resultsdir/full_correction.3.map LOCUS FILE: 05.resultsdir/05.full_correction.3.inputmatrix

# Chromosome 4 - Page 8

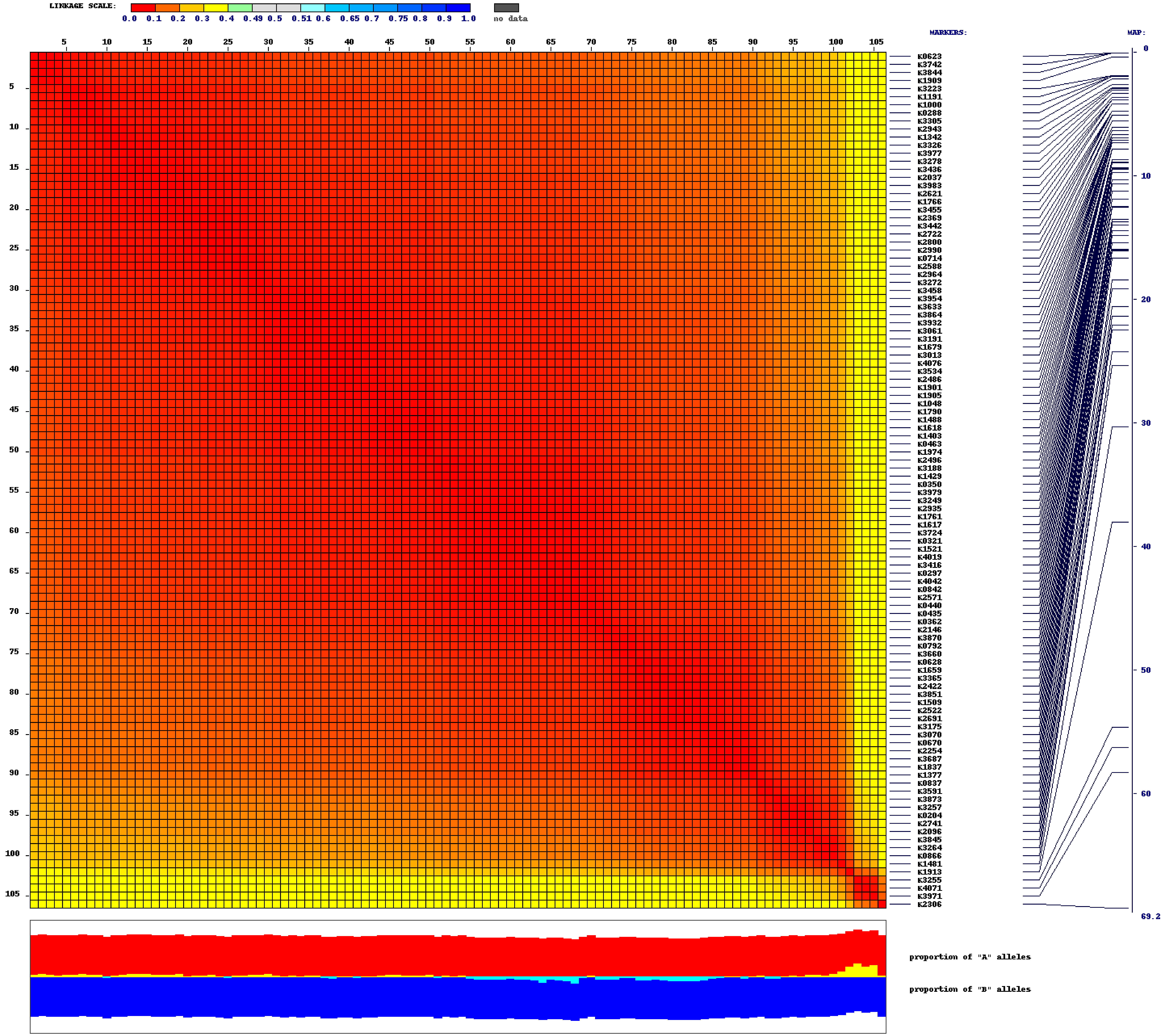


MAP: 05.resultsdir/full_correction.4.map MATRIX: 05.resultsdir/full_correction.4.pairs_CUTOFF: 0.4

COLOR SCHEME: - A  - B  - C : not A  - D : not B  - H : A+B  - no data

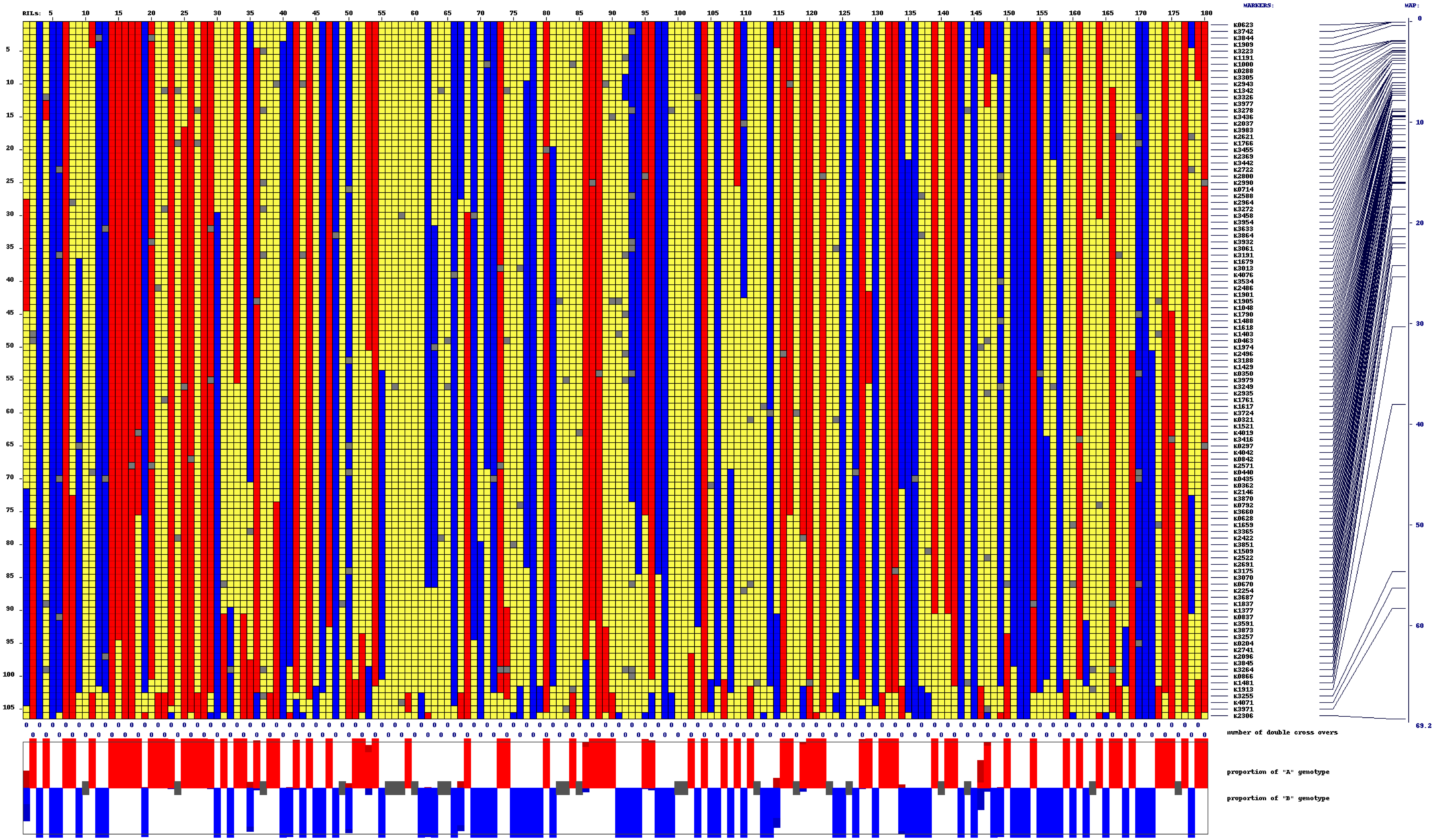


MAP: 05.resultsdir/full_correction.4.map  LOCUS FILE: 05.resultsdir/05.full_correction.4.inputmatrix

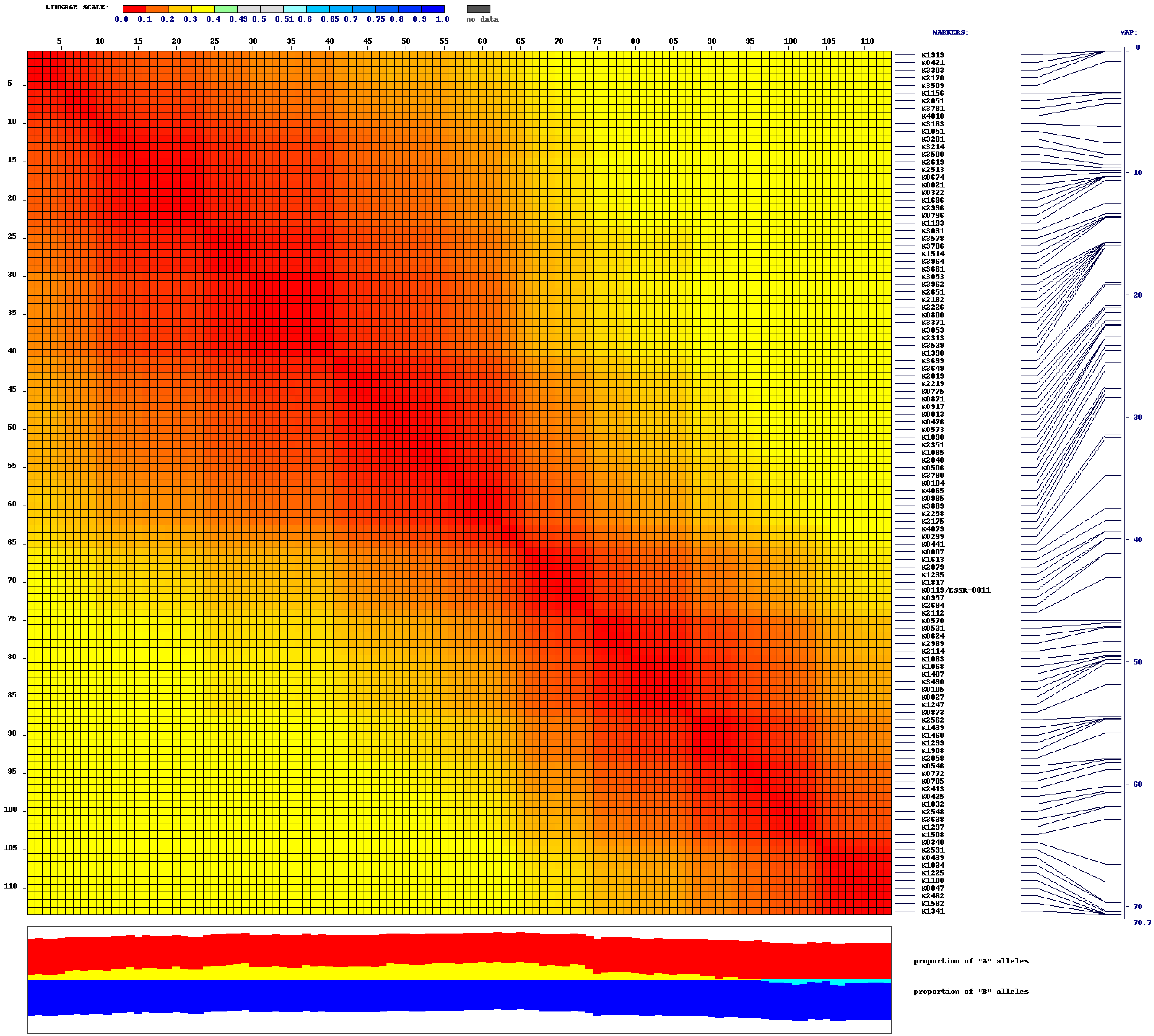


MAP: 05.resultsdir/full_correction.5.map MATRIX: 05.resultsdir/full_correction.5.pairs_CUTOFF: 0.4

COLOR SCHEME: – A – B – C : not A – D : not B – H : A+B – no data

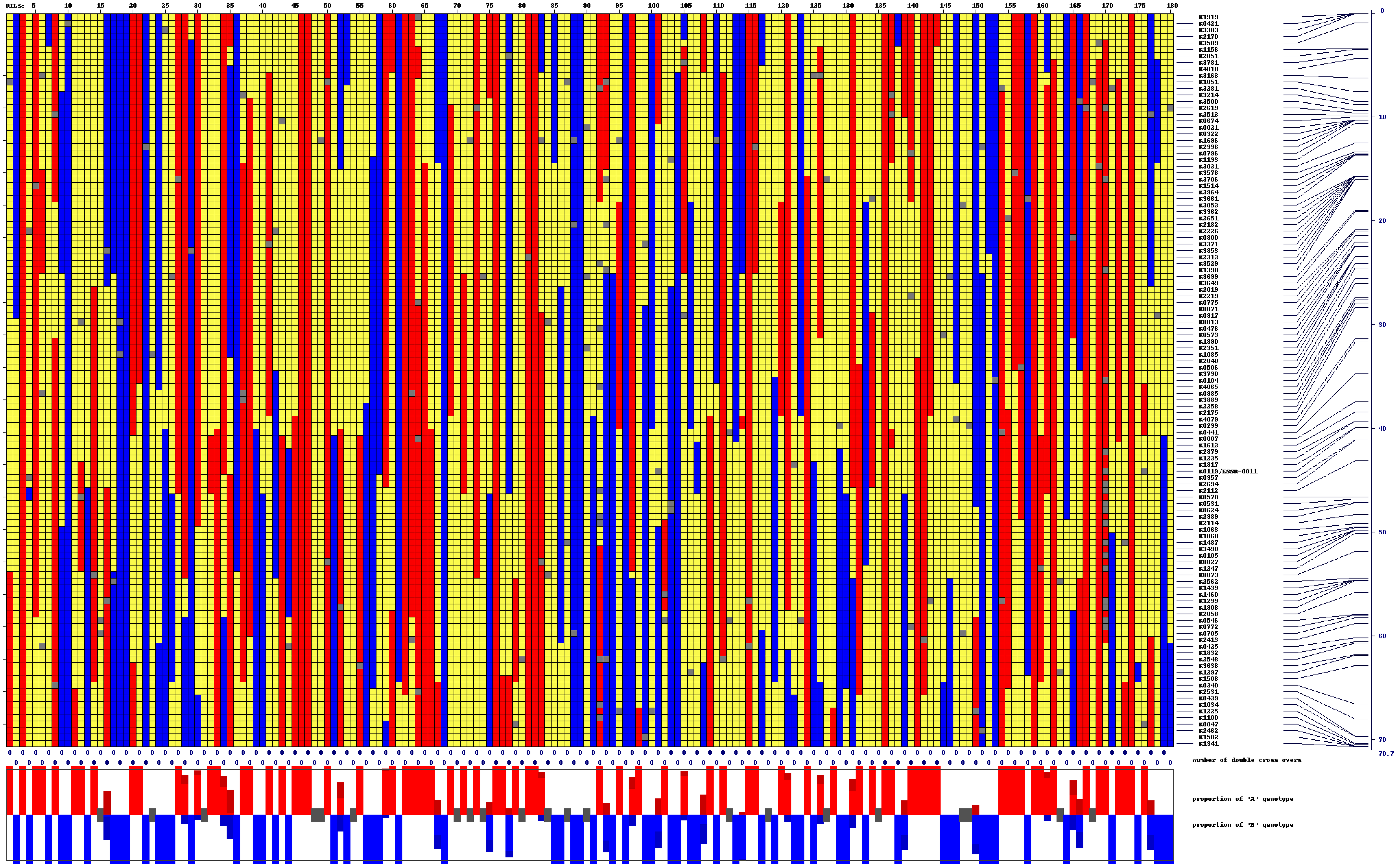


MAP: 05.resultsdir/full_correction.5.map LOCUS FILE: 05.resultsdir/05.full_correction.5.inputmatrix

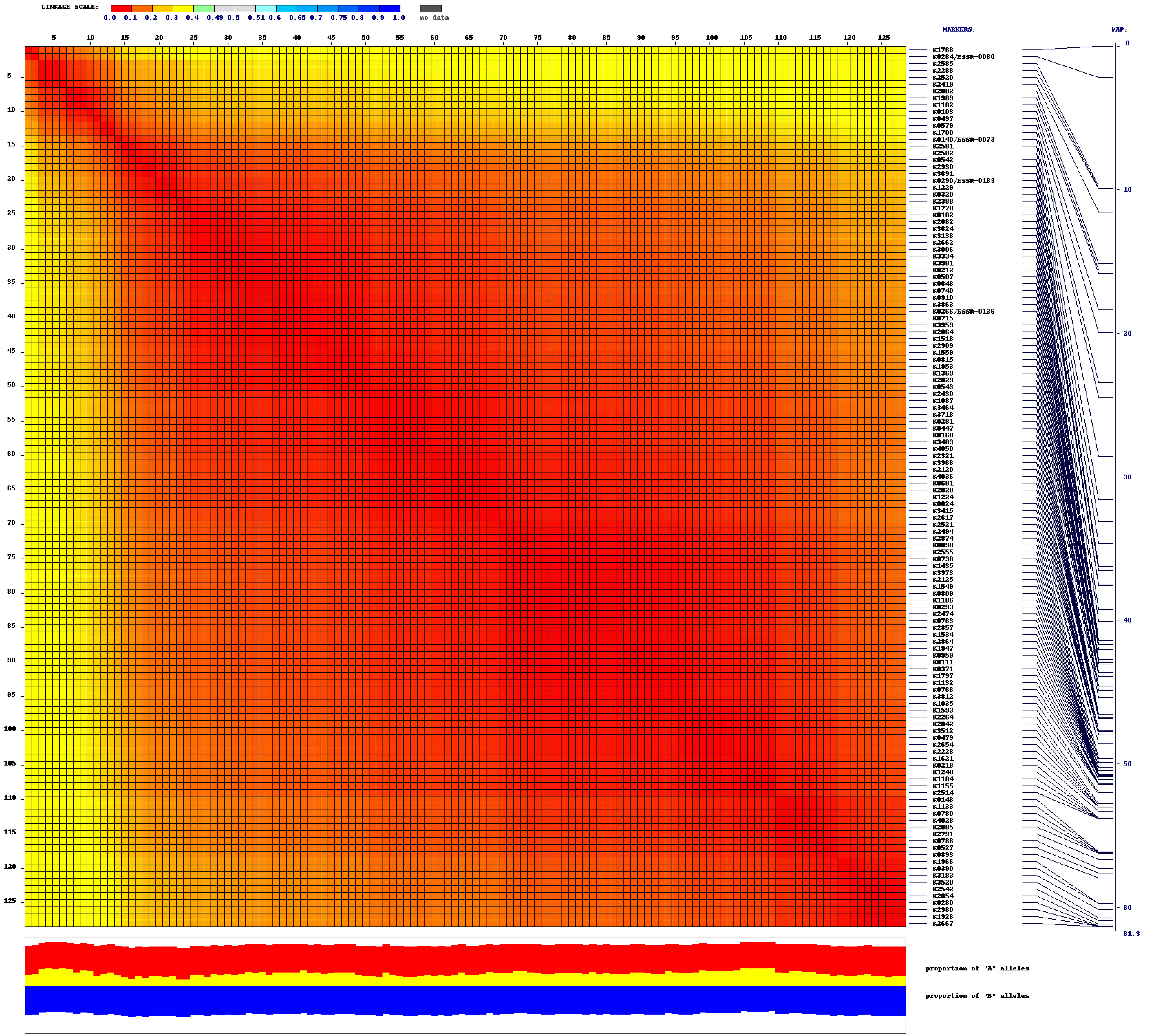


MAP: 05.resultsdir/full_correction.6.map MATRIX: 05.resultsdir/full_correction.6.pairs_all CUTOFF: 0.4

# Chromosome 6 - Page 13

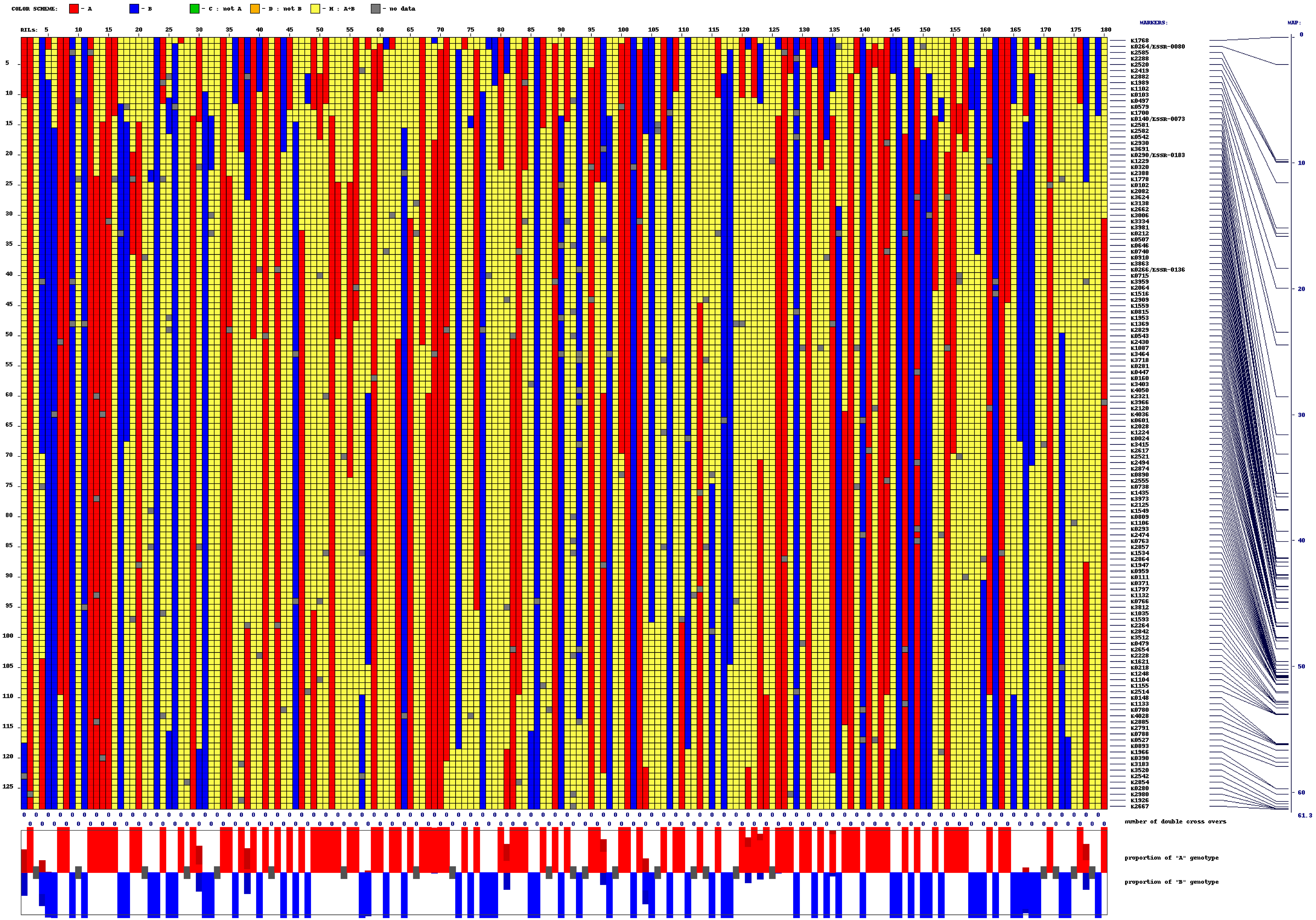


MAP: 05.resultsdir/full_correction.6.map LOCUS FILE: 05.resultsdir/05.full_correction.6.inputmatrix

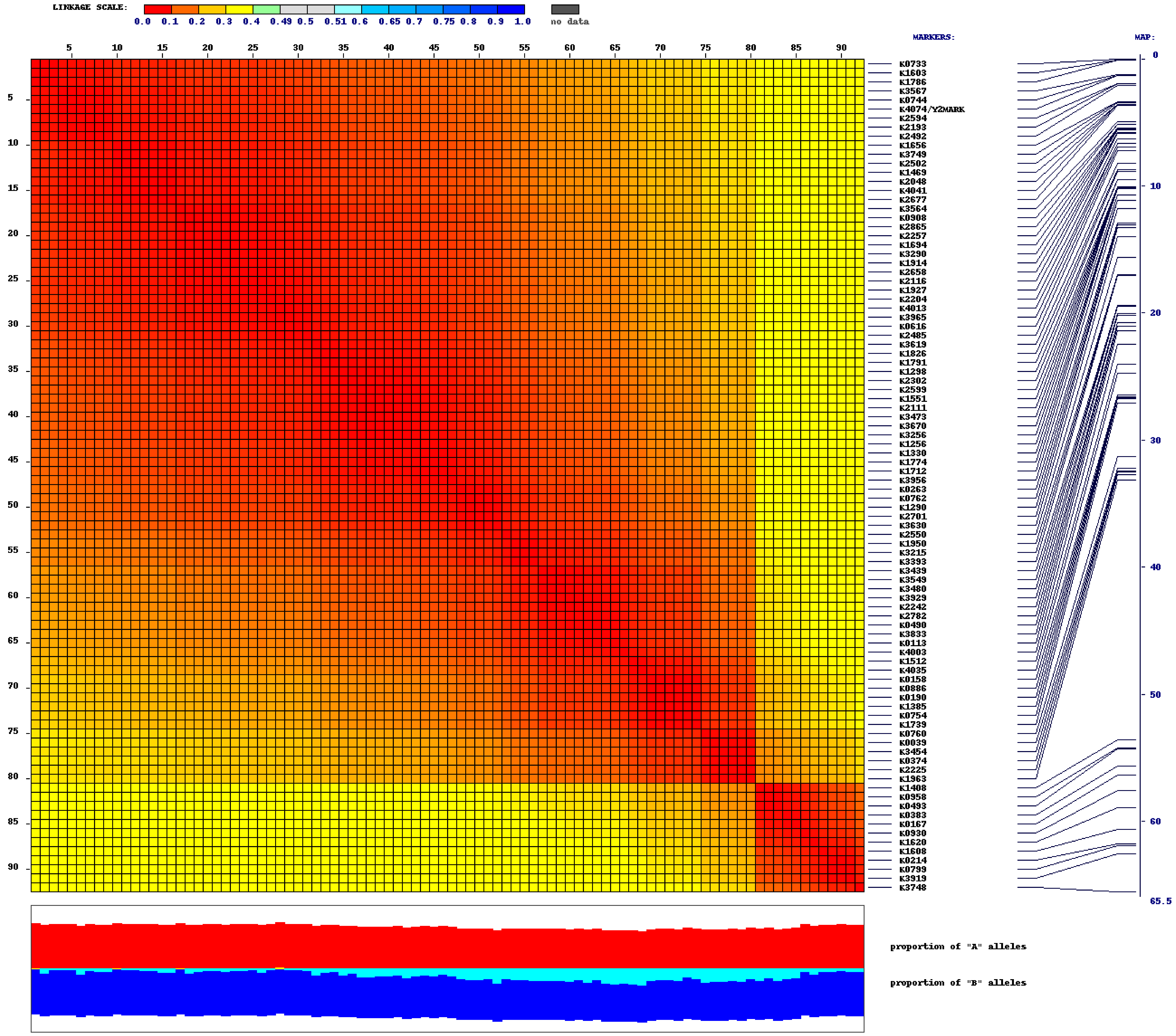


MAP: 05.resultsdir/full_correction.7.map MATRIX: 05.resultsdir/full_correction.7.pairs_[illegible]CUTOFF: 0.4

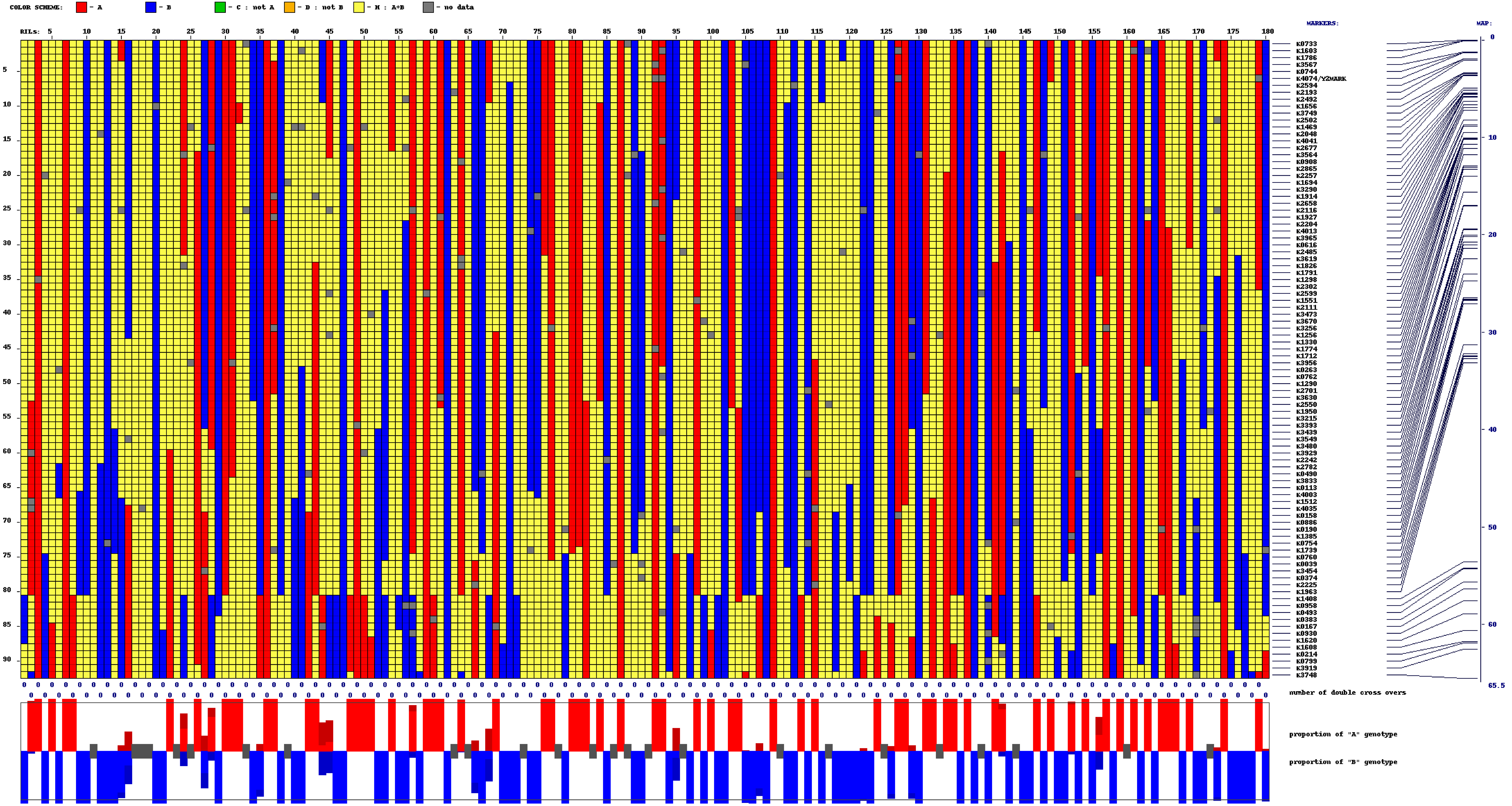


MAP: 05.resultsdir/full_correction.7.map LOCUS FILE: 05.resultsdir/05.full_correction.7.inputmatrix

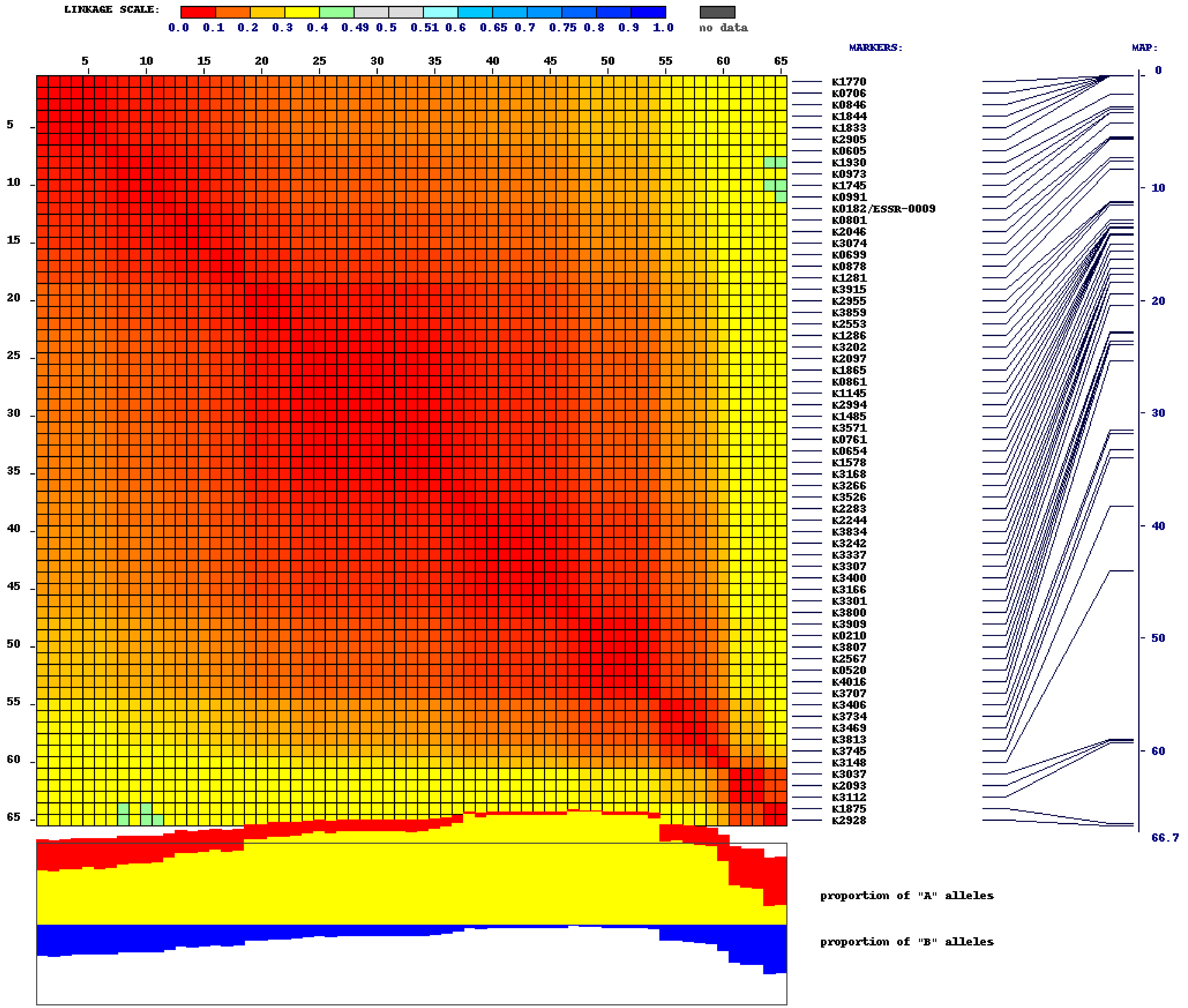


MAP: 05.resultsdir/full_correction.8.map MATRIX: 05.resultsdir/full_correction.8.pairs_[illegible]OFF: 0.4

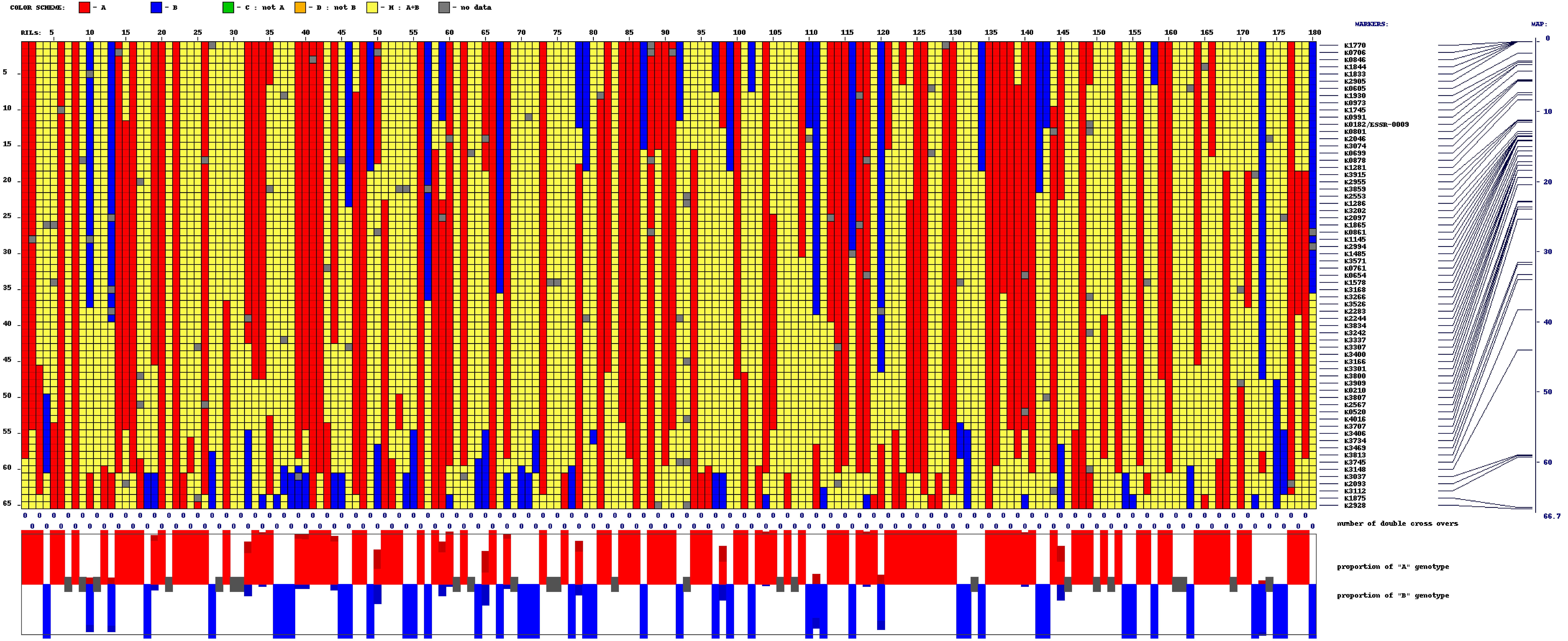


MAP: 05.resultsdir/full_correction.8.map LOCUS FILE: 05.resultsdir/05.full_correction.8.inputmatrix

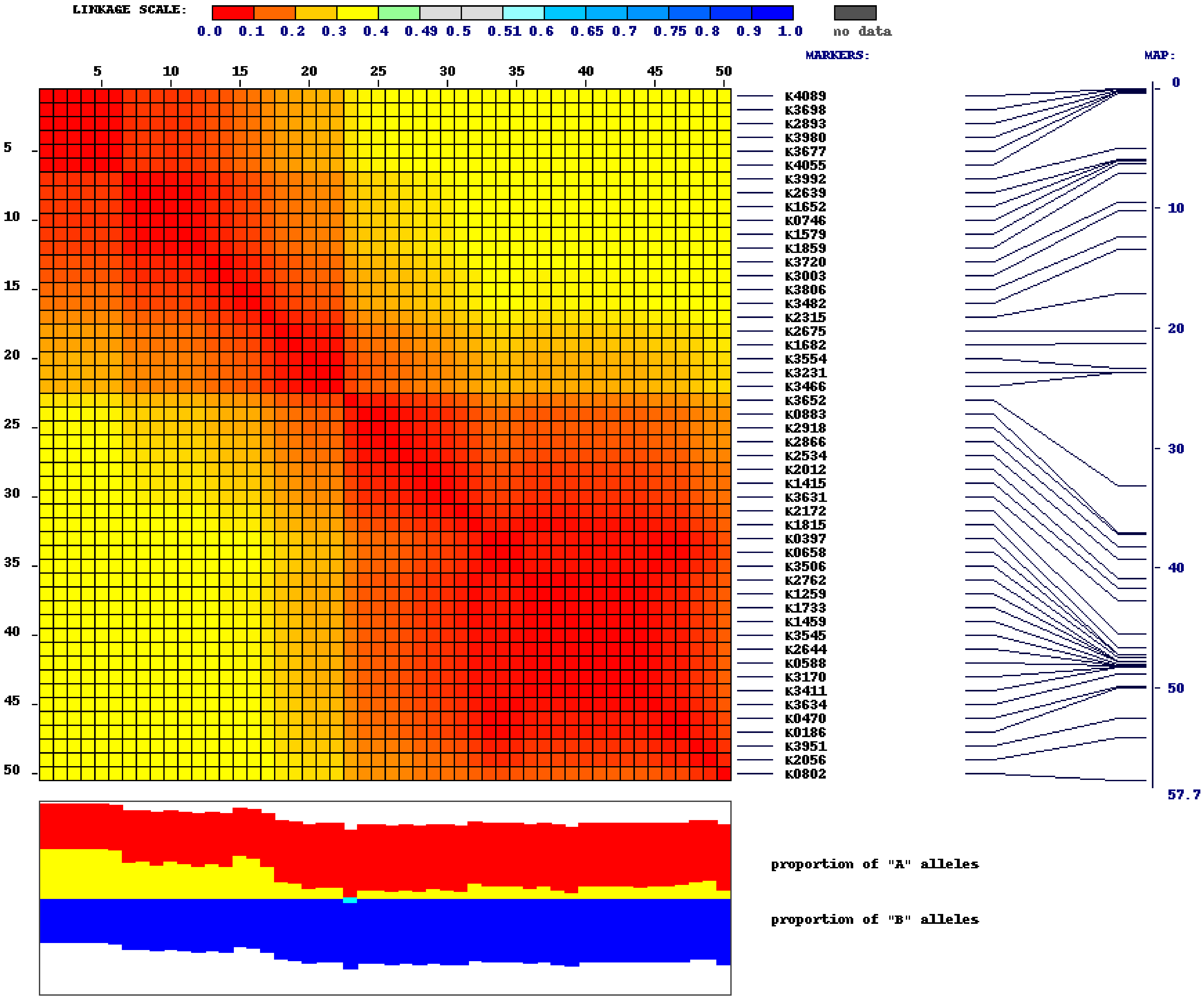


MAP: 05.resultsdir/full_correction.9.map MATRIX: 05.resultsdir/full_correction.9.pairs_[illegible]OFF: 0.4

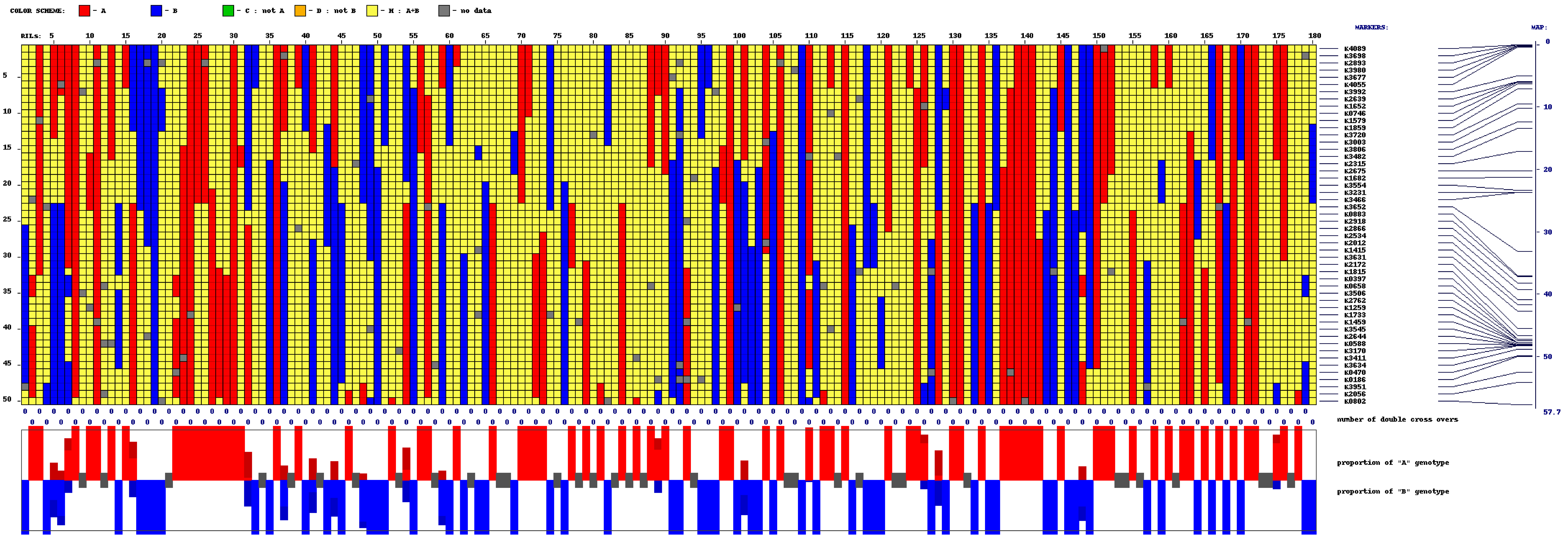


MAP: 05.resultsdir/full_correction.9.map LOCUS FILE: 05.resultsdir/05.full_correction.9.inputmatrix
